# CD8^+^ T cell transcriptional reprogramming toward TCF7-low cytotoxicity drives immune aging in viral ARDS

**DOI:** 10.1016/j.isci.2026.117774

**Published:** 2026-10-08

**Authors:** Kuan Li, Kaibinuer Ainiwaer, Licheng Song, Jinmiao Hu, Yafan Yang, Yimeng Liu, Hongwei Li, Shiqi Xu, Youyang Wang, Juan Yin, Li Li, Xing Wang, Xiaoyang Hong, Lixin Xie, Huaiyong Chen

**Affiliations:** 1 Department of Basic Medicine, Haihe Clinical School, Tianjin Medical University, Tianjin 300350, China; 2 Tianjin Key Laboratory of Lung Regenerative Medicine, Haihe Hospital, Tianjin University, Tianjin 300350, China; 3 Tianjin Institute of Respiratory Diseases, Tianjin 300350, China; 4 Senior Department of Respiratory and Critical Care Medicine, the Eighth Medical Center of Chinese PLA General Hospital, Beijing 100039, China; 5 Department of Respiratory Medicine, Haihe Clinical School, Tianjin Medical University, Tianjin 300350, China; 6 Department of Anaesthesiology, National Cancer Center/Cancer Hospital, Chinese Academy of Medical Sciences and Peking Union Medical College, Beijing 100021, China; 7 Senior Department of Pediatrics, the Seventh Medical Center of Chinese PLA General Hospital, Beijing 100700, China

**Keywords:** acute respiratory distress syndrome, immune aging, CD8^+^ effector memory T cells, granzyme B hyperactivation, TGFB1-RUNX3-PRDM1 regulatory axis

## Abstract

Viral acute respiratory distress syndrome (ARDS) exhibits distinct clinical outcomes across age groups, but the immune mechanisms underlying this disparity remain unclear. Here, we integrated single-cell transcriptomic profiling, T cell receptor repertoire analysis, serum proteomics, and functional validation to characterize age-dependent T cell remodeling in influenza-associated ARDS. Adult ARDS was characterized by reduced T cell receptor diversity, expansion of CD8^+^ effector memory T cells, and enhanced cytotoxic programs marked by increased granzyme B (GZMB) expression compared with pediatric ARDS. At the transcriptional level, adult CD8^+^ effector memory T cells displayed a TCF7-low state associated with activation of a TGFB1-related RUNX3/PRDM1 program. *In vitro* experiments further demonstrated that TGFB1 promoted cytotoxic programming in CD8^+^ effector memory T cells and increased epithelial injury in a co-culture model. These findings identify age-associated CD8^+^ T cell remodeling as a potential mechanism contributing to immune dysregulation in viral ARDS.

## Highlights

• Age-dependent CD8^+^ T cell remodeling distinguishes adult from pediatric ARDS
• Pediatric ARDS shows CD8^+^ T cell rewiring with controlled GZMB induction
• Adult TGFB1 and GZMB levels are higher at baseline and further amplified in ARDS
• TGFB1 promotes RUNX3/PRDM1-associated GZMB induction and lung epithelial apoptosis

## Graphical abstract

**Figure:**
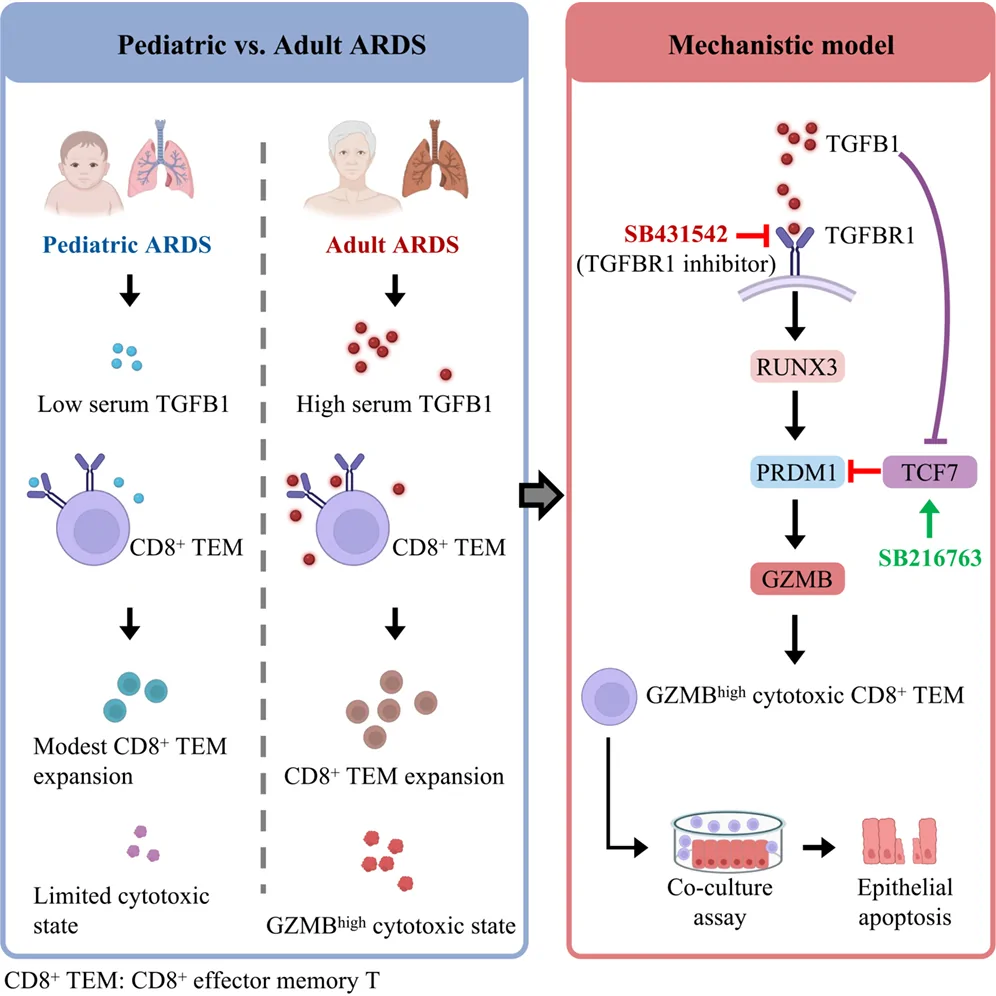

## Teaser

Health sciences; Immunity

## Introduction

Viral acute respiratory distress syndrome (ARDS) is a form of acute hypoxemic respiratory failure caused by diffuse injury to the alveolar-capillary barrier, characterized by increased pulmonary permeability, reduced lung compliance, and bilateral infiltrates.^1^ Despite substantial progress in supportive management, ARDS continues to carry a high mortality rate.^2^ Epidemiological studies indicate that adult ARDS exhibits markedly higher mortality than pediatric ARDS,^3^ suggesting the presence of age-dependent immunological differences between the two populations.

Immune cell-mediated inflammation represents the central mechanism underlying ARDS pathogenesis.^4^ During the acute phase, innate immune responses driven by neutrophils and macrophages rapidly trigger a “cytokine storm,” followed by adaptive immune activation in which T cells participate in the dynamic balance between tissue injury and repair.^5^^,^^6^^,^^7^ Among these, CD8^+^ T cells act as a double-edged sword—while clearing infected targets, they may also cause bystander injury to non-infected alveolar epithelial cells.^8^^,^^9^^,^^10^ Previous studies have shown that pediatric T cell repertoires display greater diversity and antigen responsiveness,^11^ whereas adult T cells exhibit impaired antigen recognition and dysregulated effector activity due to immune aging, thereby aggravating lung injury.^12^^,^^13^ However, whether these differences reflect changes in T cell subset abundance or a coordinated shift in CD8^+^ T cell transcriptional programs remains unclear.

Immune aging is characterized by thymic involution, imbalance between naive and memory T cell compartments, chronic antigenic stimulation, and persistent low-grade inflammation.^14^ Senescent T cells progressively acquire an effector memory phenotype, marked by loss of CD27/CD28, reduced CCR7 expression, and increased CD57 and KLRG1 expression.^15^^,^^16^ Accelerated accumulation of aged T cells in elderly individuals suggests that T cells in adult ARDS exist in a state of chronic activation coupled with functional decline, leading to immune dysregulation and tissue damage.^17^ These observations suggest that age-associated CD8^+^ T cell alterations may represent a transcriptional program shift rather than a simple expansion of effector memory subsets.

At the molecular level, the RUNX3-PRDM1 axis serves as a key pathway governing cytotoxic differentiation in CD8^+^ T cells. RUNX3 promotes the transition from central to effector memory and enhances granzyme B (GZMB) expression,^18^^,^^19^ while the transcription factor Blimp-1 (PRDM1) is essential for the differentiation and maintenance of effector and memory T cells.^20^^,^^21^ Moreover, circulating TGFB1 levels remain persistently elevated in patients with ARDS, and TGF-β has been recognized as an important pathogenic factor in this disease.^22^^,^^23^^,^^24^ TGF-β signaling enhances the expression of RUNX3 and activates its downstream cytotoxic program.^25^^,^^26^^,^^27^^,^^28^ Although these pathways have been extensively studied in CD8^+^ T cell differentiation, their role in defining disease-associated transcriptional programs in ARDS remains unclear. Together, these findings suggest that aging-associated hyperactivation of the TGF-β-RUNX3-PRDM1 axis may drive T cell cytotoxic imbalance in adult ARDS.

Collectively, these observations raise the possibility that age-dependent differences in ARDS reflect a coordinated shift in CD8^+^ T cell transcriptional programs toward a cytotoxic state-like configuration. We hypothesized that divergent outcomes between adult and pediatric ARDS arise from aging-related remodeling of T cell function and T cell receptor (TCR) clonotype diversity. Using integrated single-cell RNA and TCR sequencing, we compared T cell lineages and functional states across age groups. Adult ARDS showed expansion of CD8^+^ effector memory T cells with elevated granzyme expression, particularly GZMB, driven by the TGF-β-RUNX3-PRDM1 axis with sustained TCF7 downregulation and supported by *in vitro* validation. Collectively, this study identifies aging-dependent T cell dysfunction, impaired antigen recognition, and cytotoxic dysregulation as central immune mechanisms in adult ARDS.

## Results

### Global comparative analysis across pediatric and adult cohorts reveals reduced T cell abundance, impaired TCR diversity, and elevated granzyme activity in adult ARDS

To explore mechanisms underlying divergent outcomes of pediatric and adult ARDS, particularly the role of T cells, we analyzed peripheral blood single-cell transcriptome and TCR sequencing data from five groups: pediatric controls (*n* = 13), pediatric ARDS (*n* = 10), pediatric ARDS recovery (*n* = 10), adult controls (*n* = 12), and adult ARDS (*n* = 9) (Figure 1A and Tables S1 and S2). All ARDS patients had evidence of recent influenza virus infection based on serum IgM testing. T cells were identified using canonical markers (Figures S1A and S1B). Their proportion was reduced in pediatric ARDS versus pediatric controls and in adult ARDS versus adult controls (Figures 1B and 1C). Adult ARDS showed a decreasing trend relative to pediatric ARDS, whereas pediatric ARDS recovery exhibited a rebound (Figures 1B and 1C). Consistently, routine blood counts confirmed significantly lower absolute lymphocyte numbers in adult ARDS than pediatric ARDS (Figure 1D and Table S3), highlighting the involvement of T cells in ARDS progression across age groups.

**Figure 1:**
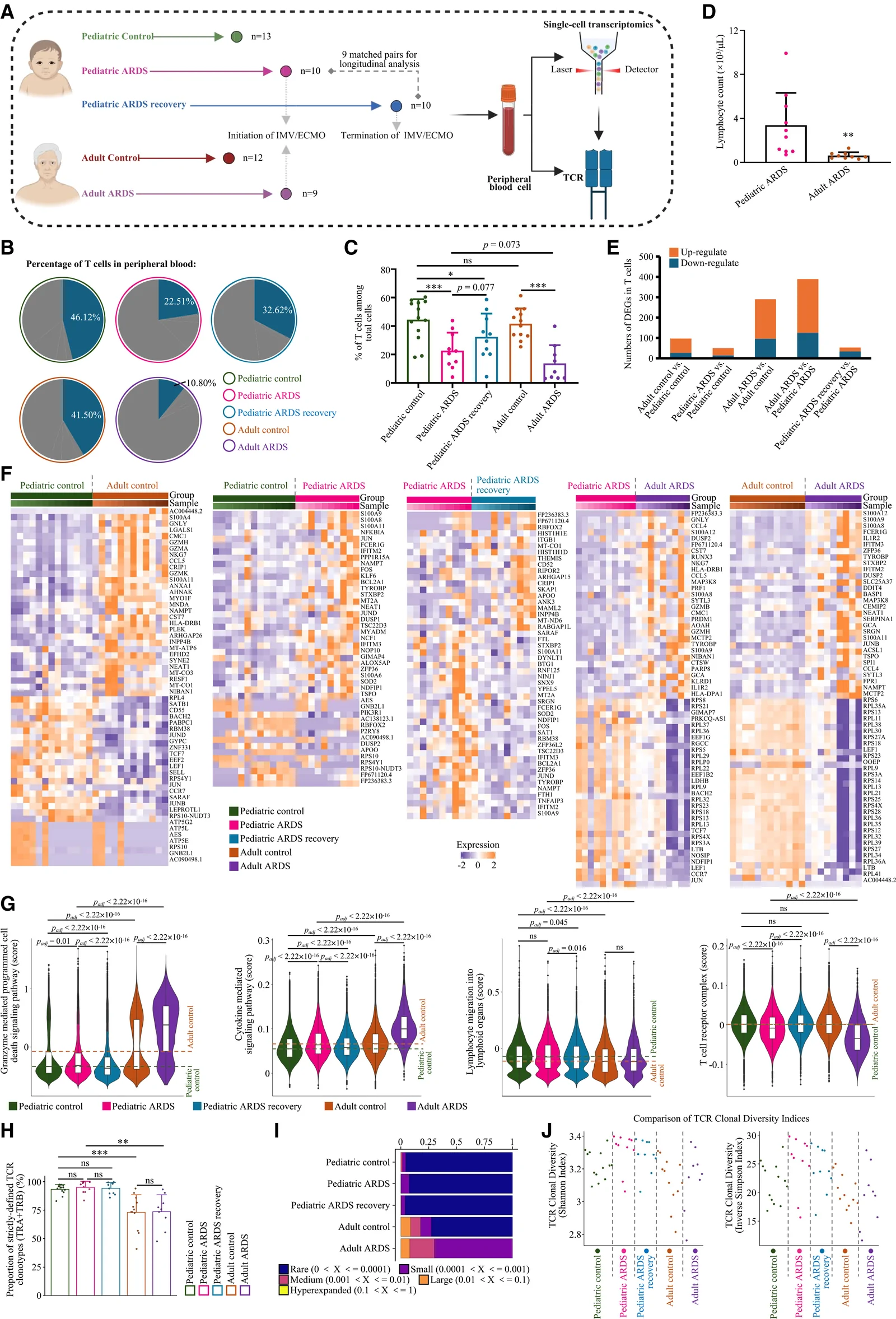
Comparative analysis of T cell abundance, TCR repertoire diversity, and granzyme pathway activity across pediatric and adult cohorts, highlighting marked disruption in adult ARDS (A) Schematic of the study design. Peripheral blood samples were collected from pediatric controls (*n* = 13), pediatric ARDS patients (*n* = 10), pediatric ARDS recovery patients (*n* = 10), adult controls (*n* = 12), and adult ARDS patients (*n* = 9). Samples were obtained at initiation and termination of invasive mechanical ventilation (IMV) or extracorporeal membrane oxygenation (ECMO). Peripheral blood mononuclear cells were subjected to single-cell transcriptome and TCR sequencing. Among pediatric ARDS and recovery samples, 9 patients had matched longitudinal samples and were included in paired analyses. (B) Proportions of T cells among peripheral blood cells across the indicated groups identified by single-cell RNA sequencing. (C) Statistical comparison of T cell fractions across groups, with *n* representing individual patient samples, showed significantly lower percentages in ARDS groups compared with controls. (D) Clinical blood counts indicating a significant reduction in absolute lymphocyte numbers in adult ARDS compared with pediatric ARDS patients. (E) Numbers of differentially expressed genes (DEGs) in each pairwise group comparison, with upregulated genes shown in orange and downregulated genes in blue. (F) Heatmaps displaying the top 30 upregulated and top 30 downregulated DEGs for each pairwise comparison (if fewer than 30, all DEGs are shown). (G) Module scores of representative T cell pathways, including granzyme-mediated cell death, cytokine signaling, lymphocyte migration into lymphoid organs, and TCR complex signaling. (H) Proportion of unique clonotypes relative to total TCR sequences, calculated under a strict clonotype definition (paired αβ chains), showing reduced values in adult ARDS patients. (I) Distribution of clonotypes according to their abundance (rare, small, medium, large, and hyperexpanded) across groups. (J) TCR clonal diversity indices (Shannon diversity and evenness), demonstrating decreased diversity in adult ARDS patients. Data are presented as mean ± SD with individual data points shown where applicable. Group comparisons were assessed by unpaired one-tailed Student’s *t* test or Wilcoxon rank-sum test, as appropriate. Module scores were compared using Wilcoxon rank-sum tests, and *p* values were adjusted using the Benjamini-Hochberg false discovery rate (FDR) correction for multiple comparisons. For conventional group comparisons, significance levels are indicated as ∗*p* < 0.05, ∗∗*p* < 0.01, ∗∗∗*p* < 0.001. For module score comparisons, adjusted *p* values (FDR) are shown.

Differential expression analysis across five pairwise comparisons (adult control vs. pediatric control, pediatric ARDS vs. pediatric control, pediatric ARDS recovery vs. pediatric ARDS, adult ARDS vs. adult control, and adult ARDS vs. pediatric ARDS) showed that adult ARDS vs. adult control and adult ARDS vs. pediatric ARDS contained the largest numbers of differentially expressed genes (DEGs) (Figure 1E). Heatmaps illustrated distinct transcriptional profiles across comparisons (Figure 1F). Granzyme-mediated cell death and cytokine-mediated signaling were elevated in adult controls relative to pediatric controls and further enhanced in adult ARDS (Figure 1G). In contrast, lymphocyte migration and TCR complex signaling were reduced in adults, with the lowest levels observed in adult ARDS (Figure 1G).

TCR repertoire analysis revealed lower unique clonotype proportions in adults than pediatric groups (Figure 1H). Pediatric groups were dominated by rare and small clonotypes, whereas adults shifted toward large and hyperexpanded clonotypes, most prominently in adult ARDS (Figure 1I). Shannon and inverse Simpson indices were also reduced in adults, with the lowest diversity in adult ARDS, indicating impaired repertoire diversity and clonal evenness (Figure 1J). Together, adult T cells exhibit reduced abundance and TCR diversity, enhanced granzyme/inflammatory activity, and attenuated migration and receptor signaling, consistent with an aging-like phenotype. In adult ARDS, diminished antigen recognition may promote peripheral bystander cytotoxicity and contribute to greater disease severity.

### Age-related remodeling of T cell subset differentiation and transcriptional programs

To further characterize T cell heterogeneity, we performed unsupervised clustering of peripheral blood T cells and identified major subsets, including CD4^+^ naive, central memory, effector memory, and regulatory T cells (Tregs); CD8^+^ naive, central memory, and effector memory T cells; and γδ T cells (Figures 2A and S2A). Pseudotime trajectory analysis revealed naive-to-memory progression in both CD4^+^ and CD8^+^ lineages (Figures 2B and 2C). In adult controls, CD4^+^ T cells exhibited increased central memory and effector memory subsets with reduced naive cells, while adult CD8^+^ T cells showed decreased naive and central memory fractions and expanded effector memory cells (Figures 2B–2G). The frequency of γδ T cells was comparable between groups (Figure 2H). Given that loss of naive T cells is a hallmark of immune aging,^14^ these findings suggest age-related remodeling of T cell differentiation dynamics.

**Figure 2:**
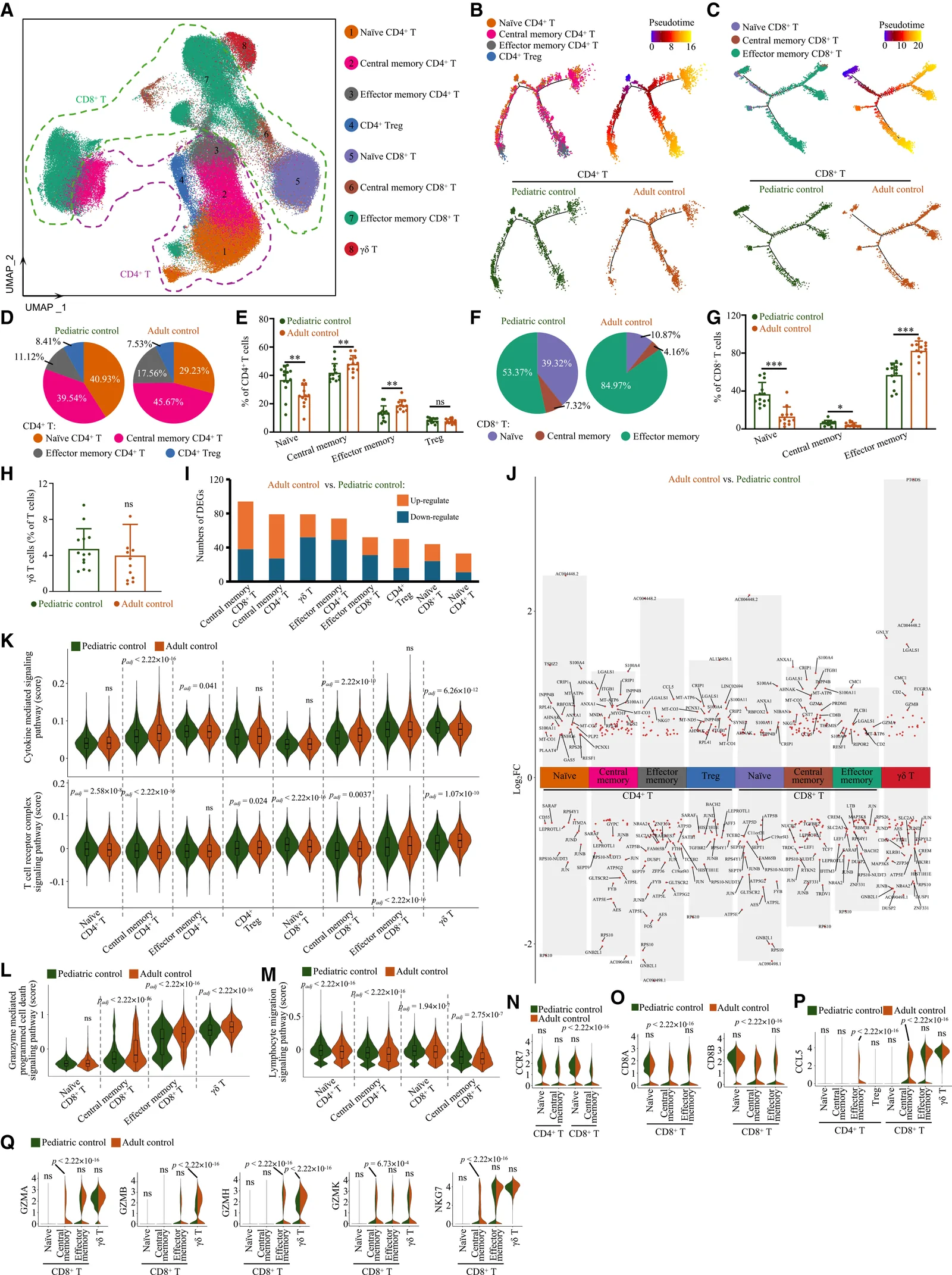
Age-related alterations in composition, differentiation, and functional programs of T cell subsets (A) UMAP projection of single-cell transcriptomes depicting major T cell subsets, including CD4^+^ naive T cells, CD4^+^ central memory T cells, CD4^+^ effector memory T cells, CD4^+^ Tregs, CD8^+^ naive T cells, CD8^+^ central memory T cells, CD8^+^ effector memory T cells, and γδ T cells. (B and C) Pseudotime trajectory analysis of CD4^+^ and CD8^+^ T cells illustrating developmental progression from naive to memory states. The upper images display trajectories colored by subset identity and pseudotime, while the lower images show trajectories stratified by pediatric and adult controls, highlighting age-related alterations in differentiation dynamics. (D and E) Pie charts (D) and bar plots (E) showing CD4^+^ T cell subset composition (naive, central memory, effector memory, and Tregs) in pediatric and adult controls based on single-cell RNA sequencing (scRNA-seq). Each dot in the bar plots represents one patient sample. Adult controls exhibited reduced naive fractions but increased central memory and effector memory proportions compared with pediatric controls. (F and G) Pie charts (F) and bar plots (G) showing CD8^+^ T cell subset composition (naive, central memory, and effector memory) in pediatric and adult controls. Adult controls were characterized by a marked reduction in CD8^+^ naive and central memory T cells, accompanied by expansion of CD8^+^ effector memory T cells. (H) Bar plot showing the frequency of γδ T cells as a percentage of total T cells in pediatric and adult controls. Each dot represents one patient sample. (I) Bar chart summarizing the number of differentially expressed genes (DEGs) in each T cell subset between adult and pediatric controls. Subsets are ranked by total DEG counts, with upregulated genes in orange and downregulated genes in blue. (J) Volcano plots displaying DEGs for each T cell subset in adult versus pediatric controls. For each subset, all DEGs were ranked by absolute log_2_ fold change, and the top 30 genes (including both upregulated and downregulated) are shown; if fewer than 30 DEGs were identified, all are displayed. (K) Violin plots showing module scores of cytokine-mediated signaling pathways (top) and T cell receptor complex signaling pathways (bottom) across T cell subsets in pediatric and adult controls. (L) Violin plots showing module scores of the granzyme-mediated programmed cell death signaling pathway in cytotoxic T cell subsets (CD8^+^ naive, CD8^+^central memory, CD8^+^ effector memory, and γδ T cells). (M) Violin plots showing module scores of the lymphocyte migration signaling pathway in homing-related T cell subsets (CD4^+^ naive, CD4^+^ central memory, CD8^+^ naive, and CD8^+^ central memory T cells). (N) Violin plots showing expression of the homing gene CCR7 in CD4^+^ and CD8^+^ naive, and central memory T cells from pediatric and adult controls. (O) Violin plots showing expression of T cell receptor complex genes CD8A and CD8B in CD8^+^ T cells from pediatric and adult controls. (P) Violin plots showing expression of cytokine gene CCL5 across T cell subsets in pediatric and adult controls. (Q) Violin plots showing expression of granzyme pathway genes (GZMA, GZMB, GZMH, GZMK, and NKG7) in cytotoxic T cell subsets (CD8^+^ naive, CD8^+^ central memory, CD8^+^ effector memory, and γδ T cells). Data in (E), (G), and (H) are shown as mean ± SD and compared using unpaired one-tailed Student’s *t* test (∗*p* < 0.05, ∗∗*p* < 0.01, ∗∗∗*p* < 0.001). Module scores in (K)–(M) were compared using Wilcoxon rank-sum tests, and *p* values were adjusted using the Benjamini-Hochberg false discovery rate (FDR) correction for multiple comparisons. For gene expression in (N)–(Q), significance was determined using the Seurat FindMarkers() function (Wilcoxon rank-sum test with Benjamini-Hochberg correction).

Transcriptomic comparison between pediatric and adult controls revealed the largest numbers of DEGs in CD8^+^ central memory T cells, CD4^+^ central memory T cells, γδ T cells, and CD4^+^ effector memory T cells (Figure 2I), with volcano plots confirming subset-specific signatures (Figure 2J). Cytokine-mediated signaling was enhanced in adult CD4^+^ central memory T cells and CD8^+^ central memory T cells but reduced in adult CD4^+^ effector memory and γδ T cells compared with pediatric controls (Figure 2K). Receptor complex signaling was increased in adult Tregs, CD8^+^ central memory, CD8^+^effector memory, and γδ T cells, whereas it was decreased in adult CD4^+^ naive, CD4^+^central memory, and CD8^+^ naive T cells (Figure 2K).

Within cytotoxic subsets, CD8^+^ central memory, CD8^+^ effector memory, and γδ T cells from adults displayed higher scores for the granzyme-mediated programmed cell death pathway (Figure 2L). In contrast, homing-related subsets (CD4^+^ naive and central memory and CD8^+^ T cells) had lower lymphocyte migration scores in adults, accompanied by downregulation of the homing receptor CCR7 (Figures 2M and 2N). Among receptor complex genes, CD8A and CD8B were reduced in adult CD8^+^ central memory T cells, while CD4 and CD3D/E/G expression remained comparable between groups (Figures 2O, S2B, and S2C). The cytokine effector CCL5 was markedly upregulated in adult CD4^+^ effector memory and CD8^+^ central memory T cells (Figure 2P). Granzyme family genes showed subset-specific increases, with GZMA, GZMK, and NKG7 elevated in adult CD8^+^ central memory T cells, GZMH increased in adult CD8^+^ effector memory, and GZMB upregulated only in adult γδ T cells (Figure 2Q). SRGN and PRF1 expression did not differ significantly between pediatric and adult controls across cytotoxic T cell subsets (Figure S2D). In summary, adult T cells exhibited reduced naive fractions, impaired homing capacity, and features of immune aging. Although several cytotoxic subsets showed increased granzyme expression, this was not accompanied by elevated GZMB expression in CD8^+^ effector memory T cells.

### CD4^+^ compositional shifts and CD8^+^ functional rewiring without GZMB upregulation in pediatric ARDS

To characterize T cells during pediatric ARDS progression, we performed pseudotime trajectory analysis. Both pediatric controls and ARDS patients displayed clear differentiation trajectories of CD4^+^ and CD8^+^ T cells from naive to memory states (Figures 2B, 2C, and 3A). Pediatric ARDS was characterized by reductions in CD4^+^ naive and effector memory T cells with increases in CD4^+^ central memory T cells (Figures 2D, 3B, and 3C). In contrast, CD8^+^ T cell subset distribution remained largely unchanged, whereas γδ T cell abundance was significantly reduced (Figures 2F and 3D–3F).

**Figure 3:**
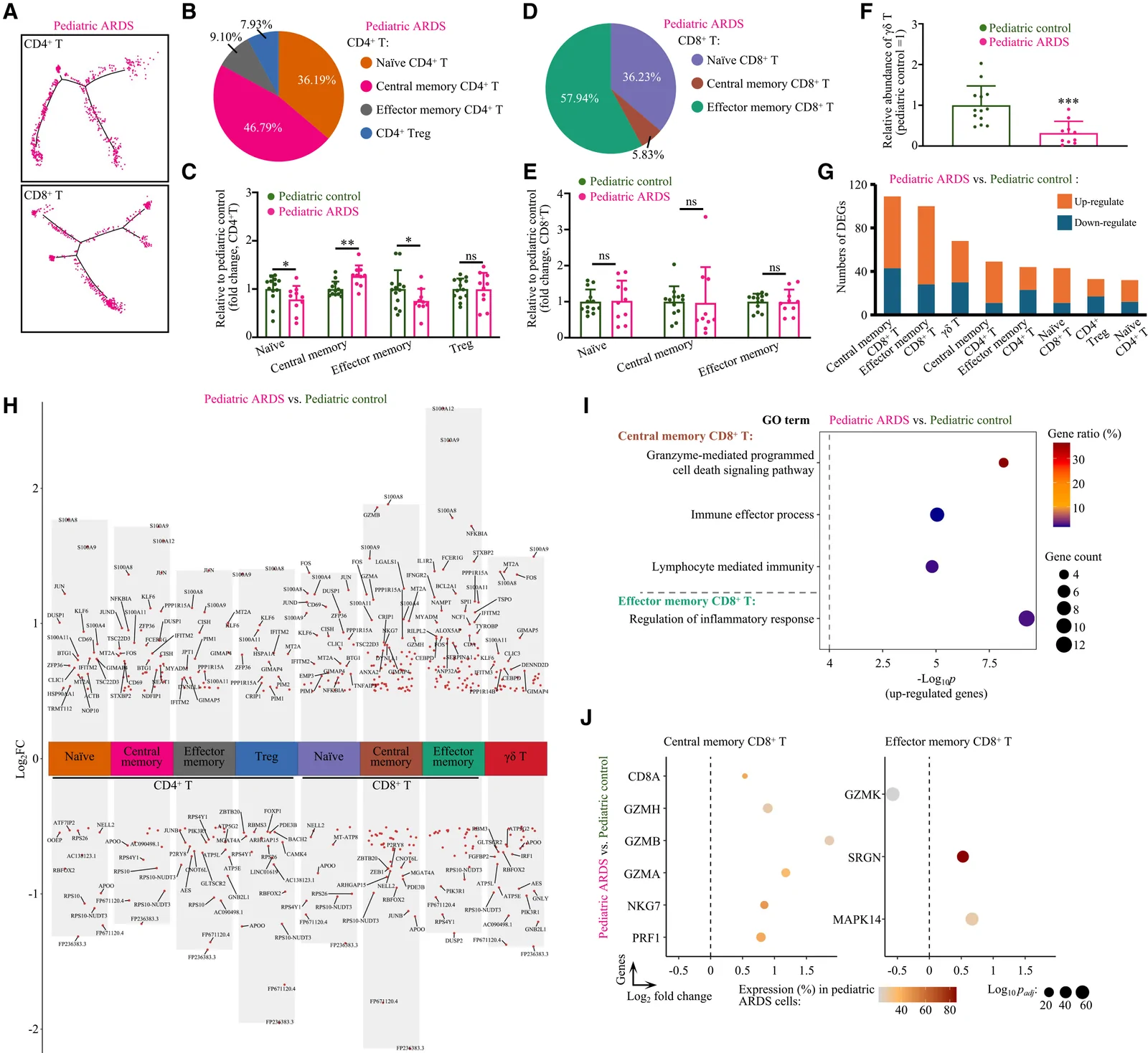
Altered CD4^+^ T cell composition and CD8^+^ T cell functional rewiring without GZMB upregulation in pediatric ARDS (A) Pseudotime trajectory analysis of CD4^+^ (top) and CD8^+^ (bottom) T cells in pediatric ARDS, illustrating differentiation from naive to memory states. (B and C) Pie charts (B) and bar plots (C) showing the distribution of CD4^+^ T cell subsets (naive, central memory, effector memory, and Tregs) in pediatric ARDS compared with pediatric controls, based on scRNA-seq classification. Bar plots display relative abundance normalized to the pediatric control group (=1), with each dot representing an individual sample. (D and E) Pie charts (D) and bar plots (E) showing the distribution of CD8^+^ T cell subsets (naive, central memory, and effector memory) in pediatric ARDS relative to pediatric controls, normalized as described above. (F) Bar plot showing the relative abundance of γδ T cells as a percentage of total T cells in pediatric ARDS versus pediatric controls, normalized to the pediatric control group (=1). Each dot represents one sample. (G) Bar chart summarizing the number of differentially expressed genes (DEGs) in each T cell subset between pediatric ARDS and controls, ranked by total DEG counts, with upregulated genes in orange and downregulated genes in blue. (H) Volcano plots displaying DEGs for each T cell subset in pediatric ARDS versus controls. For each subset, DEGs were ranked by absolute log_2_ fold change, and the top 30 are shown; if fewer than 30 DEGs were identified, all are displayed. (I) Dot plots showing Gene Ontology (GO) enrichment analysis of DEGs in pediatric ARDS versus controls. Dot size indicates the number of DEGs per pathway, while color intensity reflects the gene ratio. (J) Dot plots displaying representative DEGs in central memory and CD8^+^ effector memory T cells from pediatric ARDS versus controls. Dot size represents statistical significance (−log_10_ adjusted *p* value), and color intensity denotes the percentage of cells expressing the gene. Data in (C), (E), and (F) are shown as mean ± SD and compared using unpaired one-tailed Student’s *t* test (∗*p* < 0.05, ∗∗*p* < 0.01, ∗∗∗*p* < 0.001). For (I), GO enrichment analysis was performed using a cumulative hypergeometric test. Differential gene expression in (J) was assessed using the Wilcoxon rank-sum test with Benjamini-Hochberg correction.

At the transcriptional level, DEG analysis revealed pronounced changes in CD8^+^ central memory and effector memory T cells (Figures 3G and 3H). Upregulated genes in CD8^+^ central memory T cells were enriched in granzyme-mediated programmed cell death and immune effector pathways, whereas those in CD8^+^ effector memory T cells were enriched in inflammatory response pathways (Figure 3I). CD8^+^ central memory T cells showed increased expression of cytotoxic molecules (PRF1, NKG7, GZMA, GZMB, and GZMH) and reduced CD8A, whereas CD8^+^ effector memory T cells upregulated cytotoxic genes GZMK and SRGN, as well as the inflammatory mediator MAPK14 (Figure 3J). Importantly, GZMB was not aberrantly elevated in CD8^+^ effector memory T cells. Collectively, pediatric ARDS exhibits CD4^+^ compositional remodeling and CD8^+^ transcriptional rewiring without excessive GZMB induction, consistent with functional adaptation rather than uncontrolled cytotoxic activation.

### Adult ARDS progression is characterized by expansion of CD8^+^ effector memory T cells and upregulation of GZMB

To investigate T cell alterations in adult ARDS, we first examined their differentiation trajectories. Both CD4^+^ and CD8^+^ T cells retained clear naive-to-memory trajectories comparable to controls (Figures 2B, 2C, and 4A). Adult ARDS patients showed reduced CD4^+^ naive T cells, decreased CD4^+^ effector memory T cells, and increased CD4^+^ central memory T cells (Figures 2D, 4B, and 4C). In the CD8^+^ T cell compartment, naive cells declined, central memory cells were reduced, and effector memory cells were markedly expanded (Figures 2F, 4D, and 4E). γδ T cell abundance was also decreased (Figure 4F). Transcriptomic profiling revealed pronounced changes in CD8^+^ central memory and effector memory T cells (Figures 4G and 4H). Gene Ontology (GO) analysis indicated enrichment of immune effector, inflammatory, and cytotoxic pathways, whereas downregulated genes were associated with T cell migration and TCR signaling (Figure 4I). CD4^+^ central memory, effector memory, and Treg cells upregulated inflammatory mediators (MAPK14, CCL4, and FAS), while CD4^+^ naive T cells exhibited reduced CD3D expression (Figure 4J).

**Figure 4:**
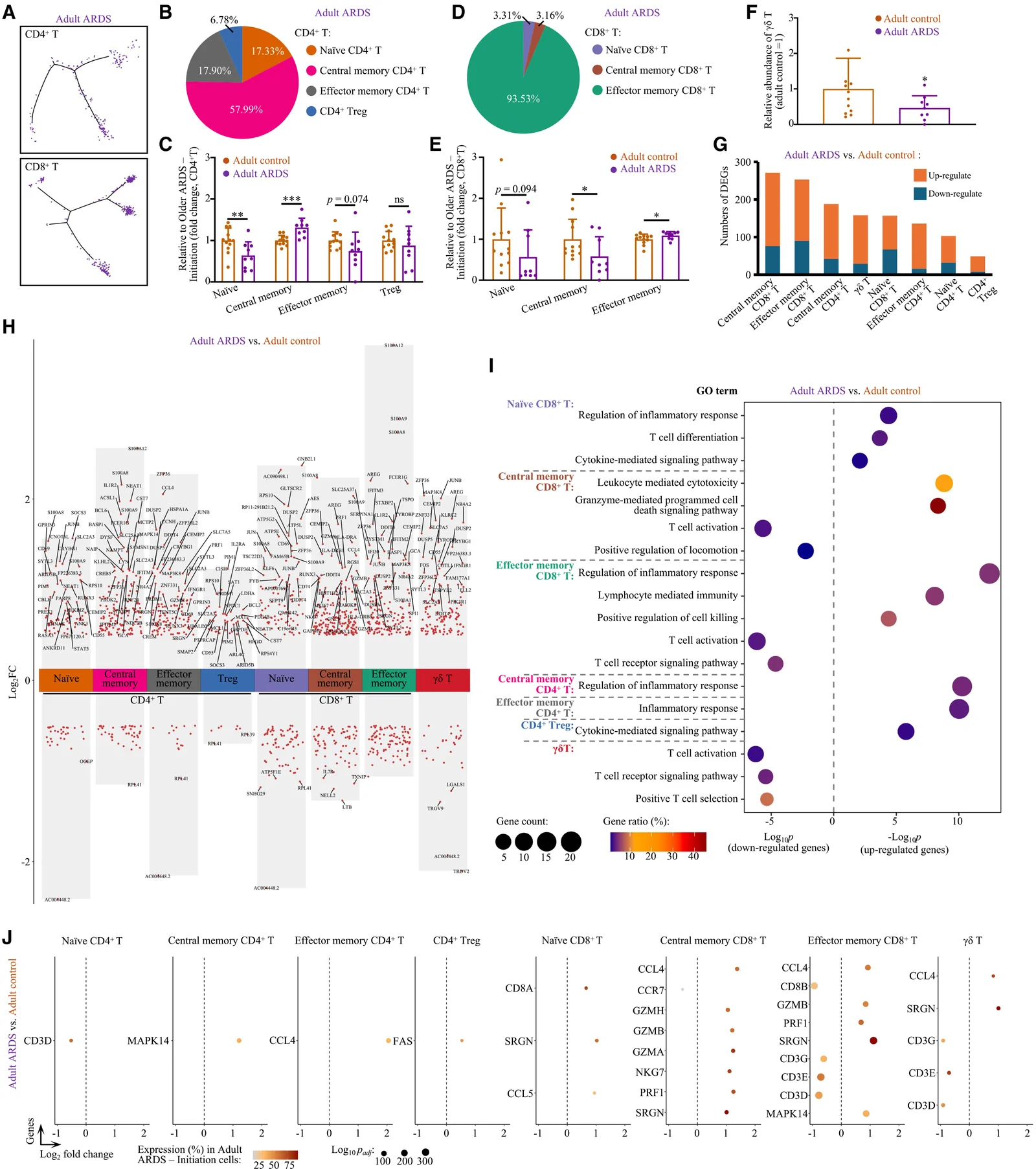
Altered distribution of CD4^+^ and CD8^+^ T cell subsets in adult ARDS progression, accompanied by an enhanced cytotoxic profile of CD8^+^ T cells (A) Pseudotime trajectory analysis of CD4^+^ (top) and CD8^+^ (bottom) T cells illustrating differentiation from naive to memory states in adult ARDS patients. (B and C) CD4^+^ T cell subsets in adult ARDS patients. (B) Pie chart showing the distribution of naive, central memory, effector memory, and Treg subsets within adult ARDS. (C) Bar plots comparing normalized proportions of CD4^+^ T cell subsets between adult ARDS and adult controls, with adult controls set as 1. Each dot represents one patient sample. (D and E) CD8^+^ T cell subsets in adult ARDS patients. (D) Pie chart showing the distribution of naive, central memory, and effector memory subsets within adult ARDS. (E) Bar plots comparing normalized proportions of CD8^+^ T cell subsets between adult ARDS and adult controls, normalized as above. (F) Bar plots showing the frequency of γδ T cells as a percentage of total T cells in adult ARDS versus controls, normalized to pediatric controls = 1. Each dot represents one sample. (G) Bar charts summarizing the number of differentially expressed genes (DEGs) across T cell subsets in adult ARDS versus controls. Subsets are ranked by total DEG counts, with upregulated genes shown in orange and downregulated in blue. (H) Volcano plots showing DEGs for each T cell subset in adult ARDS versus controls. Genes are ranked by absolute log_2_ fold change, and the top 30 are displayed; if fewer than 30 DEGs were detected, all are shown. (I) Dot plots of Gene Ontology (GO) enrichment analysis for DEGs in adult ARDS versus controls. Dot size indicates the number of DEGs per pathway, and color intensity reflects the gene ratio. (J) Dot plots displaying expression of representative DEGs across T cell subsets. Dot size represents statistical significance (−log_10_ adjusted *p* value), and color intensity denotes the percentage of cells expressing the gene. Data in (C), (E), and (F) are shown as mean ± SD and compared using unpaired one-tailed Student’s *t* test (∗*p* < 0.05, ∗∗*p* < 0.01, ∗∗∗*p* < 0.001). For (I), GO enrichment was assessed using a cumulative hypergeometric test. For (J), differential gene expression was determined using the Wilcoxon rank-sum test with Benjamini-Hochberg correction.

Among CD8^+^ T cell subsets, central memory cells demonstrated increased SRGN, PRF1, NKG7, GZMA, GZMB, GZMH, CCL4, and reduced CCR7 expression, while effector memory cells exhibited elevated SRGN, PRF1, GZMB, MAPK14, CCL4, with downregulation of CD3D/E/G and CD8B (Figure 4J). This increase in GZMB expression was further clarified by subsequent CD8^+^ effector memory T cell reclustering, which identified a distinct GZMB-high cytotoxic subset rather than a uniform increase across all effector memory cells (Figures 8E–8H). Similar TCR gene downregulation was observed in γδ T cells (Figure 4J).

Together, adult ARDS features extensive T cell compositional remodeling and transcriptional reprogramming of cytotoxic and inflammatory pathways, leading to excessive GZMB activation. Concomitant downregulation of TCR complex genes suggests impaired antigen recognition, potentially driving uncontrolled bystander killing and increasing tissue injury risk.

### Comparative analysis reveals aging features and dysregulated cytotoxicity in adult versus pediatric ARDS

To investigate age-related T cell remodeling in ARDS, we compared composition and transcriptional profiles of peripheral blood T cells from adult and pediatric patients. In CD4^+^ T cells, adult ARDS patients showed reduced naive cells and increased central memory cells versus pediatric ARDS (Figure 5A). In CD8^+^ T cells, naive subsets were decreased, central memory cells showed a downward trend (*p* = 0.057), and effector memory cells were markedly expanded (Figure 5B). γδ T cell frequency was comparable between the two groups (Figure 5C). Transcriptomic analysis revealed widespread differences, with the largest changes occurring in CD4^+^ central memory T cells and both CD8^+^ central and effector memory T cells (Figures 5D and 5E). Module scoring demonstrated higher cytokine-mediated signaling across all T cell subsets in adult ARDS, whereas receptor complex signaling was reduced in nearly all subsets except Tregs (Figure 5F). Granzyme-mediated programmed cell death scores were elevated in cytotoxic subsets (CD8^+^ naive, central memory, effector memory T cells, and γδ T cells) from adult ARDS patients Figure 5G), whereas lymphocyte migration scores showed no significant differences across T cell subsets after multiple-testing correction (Figure 5H). Representative DEGs further supported these patterns: cytokine- and inflammation-related genes (*MAPK14*, *CCL5*, and *FAS*) were upregulated in CD4^+^ memory subsets, Tregs, and major CD8^+^ subsets; TCR complex genes (*CD3D*, *CD3E*, and *CD3G*) were downregulated in CD4^+^ naive, CD8^+^ central memory, CD8^+^ effector memory, and γδ T cells, with *CD8B* also decreased in CD8^+^ central memory T cells; cytotoxic molecules (*SRGN*, *PRF1*, *NKG7*, *GZMA*, *GZMB*, *GZMH*, and *GZMK*) were increased in cytotoxic subsets; and CCR7 was downregulated in CD4^+^ and CD8^+^ central memory T cells (Figure 5I). In summary, adult ARDS exhibits reduced naive T cell frequencies, expansion of CD8^+^ effector memory T cells, impaired homing capacity, and enhanced cytotoxic programs. Notably, elevated GZMB expression in CD8^+^ effector memory T cells, together with reduced TCR-associated features, characterized a cytotoxic and dysfunctional T cell state in adult ARDS.

**Figure 5:**
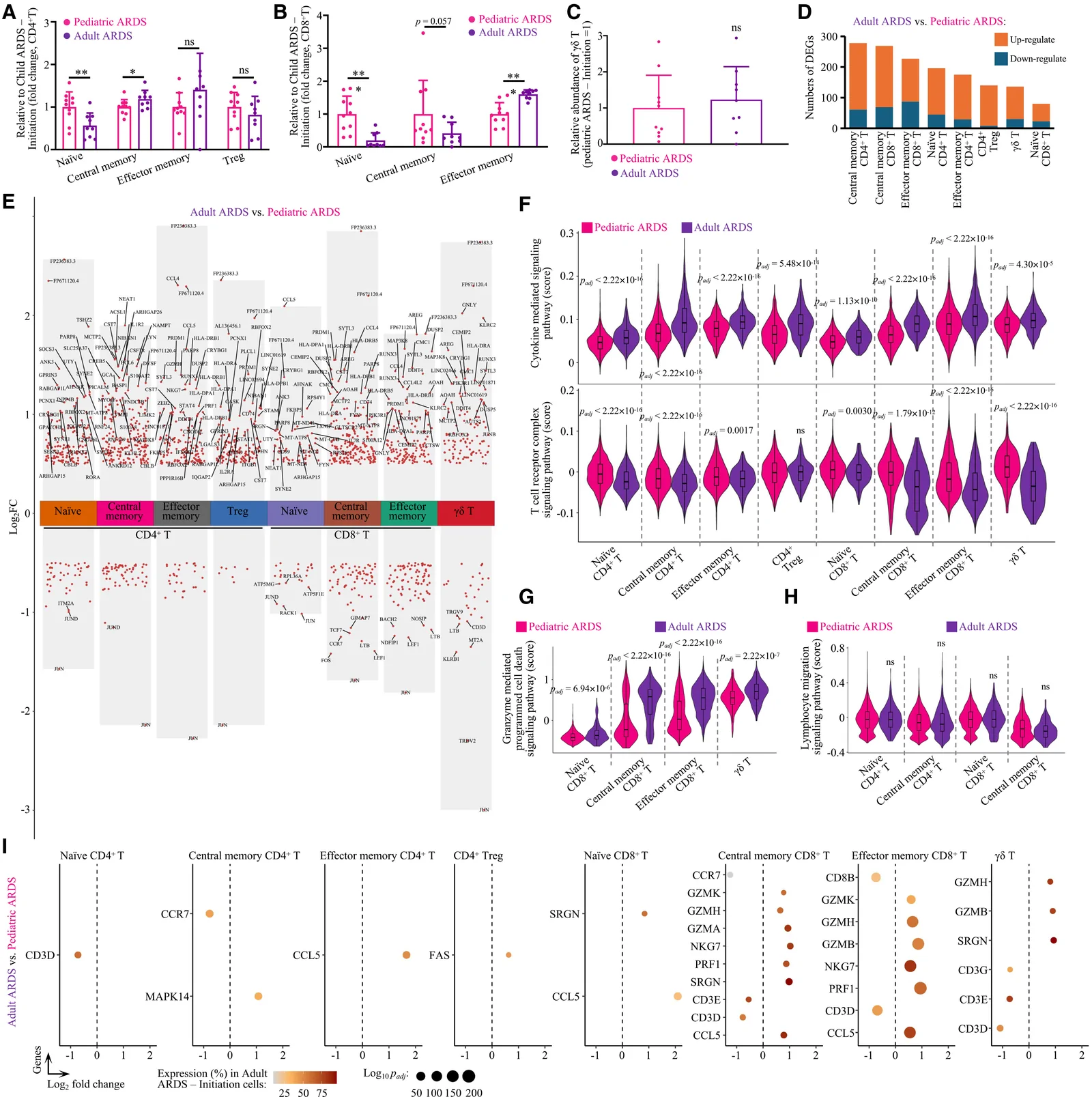
Comparative remodeling of T cell subsets and transcriptional programs in adult versus pediatric ARDS (A) Bar plots showing the distribution of CD4^+^ T cell subsets (naive, central memory, effector memory, and Tregs) in adult versus pediatric ARDS. For each individual, subset abundance was expressed as the proportion of the subset among total CD4^+^ T cells, and group values were scaled by setting the pediatric ARDS group to 1. Each dot represents one patient sample. (B) Distribution of CD8^+^ T cell subsets (naive, central memory, and effector memory) in adult versus pediatric ARDS. For each individual, subset abundance was expressed as the proportion of the subset among total CD8^+^ T cells, and group values were scaled by setting the pediatric ARDS group to 1. Each dot represents one patient sample. (C) Relative proportion of γδ T cells among total T cells in adult versus pediatric ARDS. For each individual, the proportion of γδ T cells was calculated among total T cells, and group values were scaled by setting the pediatric control group to 1. Each dot represents one patient sample. (D) Summary of differentially expressed genes (DEGs) in adult versus pediatric ARDS across T cell subsets, ranked by the total number of DEGs. Upregulated genes are shown in orange, downregulated genes in blue. (E) Volcano plots showing the top 30 DEGs (by absolute log_2_ fold change) for each T cell subset in adult versus pediatric ARDS. All DEGs are shown when fewer than 30 were detected. (F) Violin plots of module scores for cytokine-mediated signaling pathways (top) and cell receptor complex signaling pathways (bottom) across T cell subsets in adult versus pediatric ARDS. (G) Violin plots of granzyme-mediated programmed cell death pathway scores in cytotoxic subsets (CD8^+^ naive, CD8^+^ central memory, CD8^+^ effector memory, and γδ T cells) from adult and pediatric ARDS patients. (H) Violin plots of lymphocyte migration pathway scores in homing-related subsets (CD4^+^ naive, CD4^+^ central memory, CD8^+^ naive, and CD8^+^ central memory T cells) in adult and pediatric ARDS. (I) Dot plots of representative DEGs in adult versus pediatric ARDS across T cell subsets. Dot size denotes statistical significance (−log_10_ adjusted *p* value), and color intensity represents the percentage of cells expressing the indicated gene. Data in (A)–(C) are shown as mean ± SD and compared using unpaired one-tailed Student’s *t* test (∗*p* < 0.05, ∗∗*p* < 0.01, ∗∗∗*p* < 0.001). Module scores in (F)–(H) were compared using Wilcoxon rank-sum tests, and *p* values were adjusted using the Benjamini-Hochberg false discovery rate (FDR) correction for multiple comparisons. DEG significance in (I) was determined using the Seurat FindMarkers() function (Wilcoxon rank-sum test with Benjamini-Hochberg correction).

### Patient-level analysis reveals age-dependent loss of memory-associated programs in CD8^+^ effector memory T cells

To determine whether transcriptional differences between adult and pediatric ARDS reflected baseline age-related immune differences or distinct responses to ARDS across age groups, we performed a patient-level pseudobulk interaction analysis of CD8^+^ effector memory T cells, with each patient treated as an independent biological replicate. Using a DESeq2 model incorporating age, disease status, and the age × disease interaction term, we identified genes whose responses to ARDS differed between adults and children (Figure 6A). Among representative genes, CCR7 showed the strongest interaction effect, with a greater reduction in adult ARDS (Figure 6B). Patient-level analysis confirmed reduced CCR7 expression in CD8^+^ effector memory T cells from adult ARDS patients (Figure 6C). We further assessed a TCF7-associated memory module score and observed reduced memory-associated transcriptional programs in adult ARDS, with a significant age × disease interaction (Figure 6D). These findings indicate that adult ARDS is associated with greater disruption of memory-associated CD8^+^ effector memory T cell programs in an age-dependent manner.

**Figure 6:**
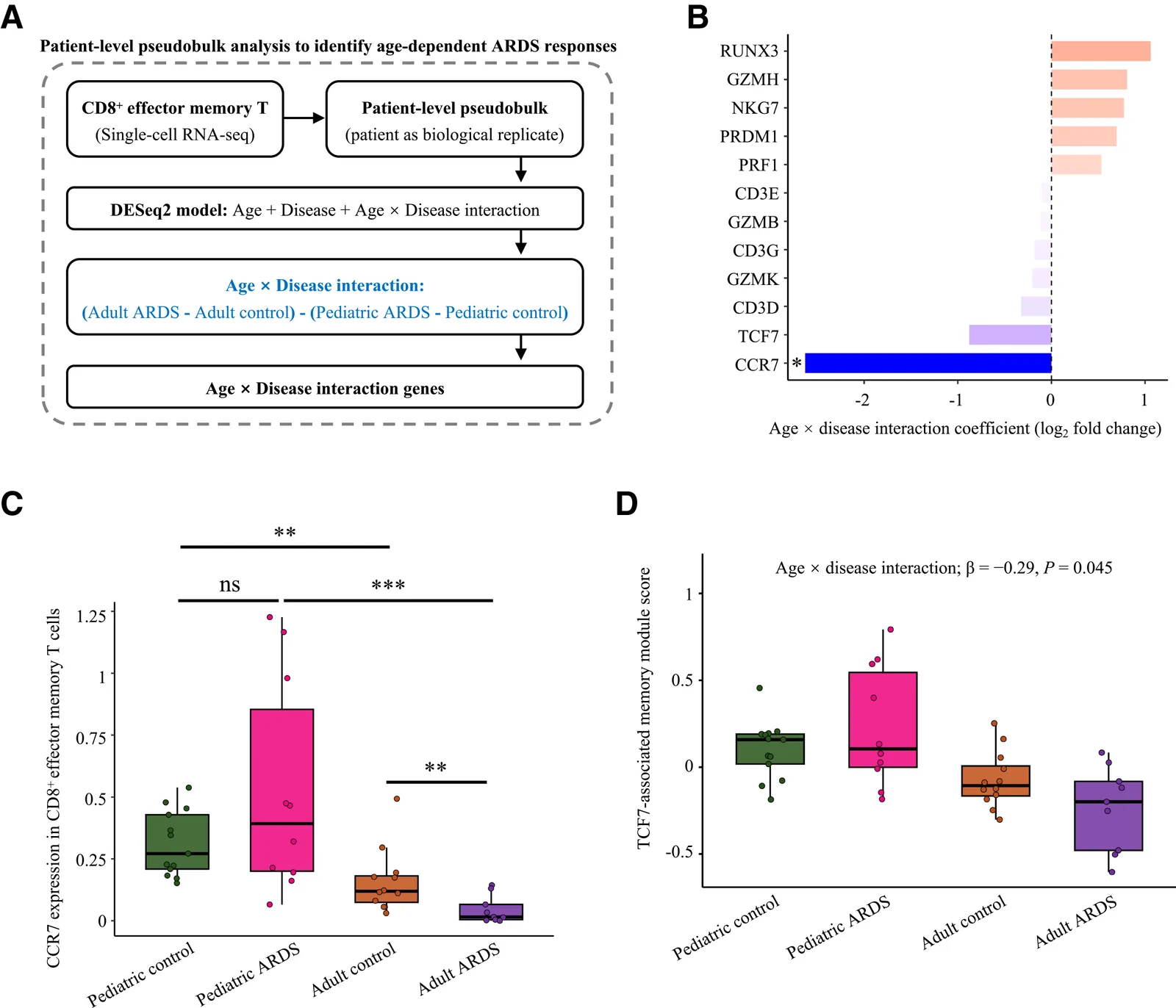
Patient-level analysis identifies age-dependent remodeling of CD8^+^ effector memory T cells (A) Workflow of the patient-level pseudobulk analysis. (B) Representative genes from the age × disease interaction analysis in effector memory CD8^+^ T cells. Interaction coefficients (β) are shown on the *x* axis. (C) Patient-level CCR7 expression in effector memory CD8^+^ T cells. CCR7 expression values were averaged across cells from each patient. Each dot represents one patient. (D) Patient-level TCF7-associated memory module score in effector memory CD8^+^ T cells. Scores were calculated using a predefined TCF7-associated memory gene signature. The age × disease interaction was assessed by linear regression. Statistical analyses were performed at the patient level. Interaction-associated genes in (B) were identified using DESeq2 with Benjamini-Hochberg correction. The age × disease interaction in (D) was evaluated using linear regression including age, disease status, and the interaction term. Each patient was considered an independent biological replicate. In (B), asterisks indicate Benjamini–Hochberg-adjusted *p* < 0.05. In (C) and (D), box plots show the median and interquartile range. Pairwise comparisons in (C) were performed using two-sided Wilcoxon rank-sum tests (ns, *p* ≥ 0.05; ∗∗*p* < 0.01; ∗∗∗*p* < 0.001), and the indicated β and *p* value in (D) correspond to the age × disease interaction term.

### Attenuated cytotoxic activation in pediatric ARDS recovery

To assess immune remodeling during pediatric ARDS recovery, we compared peripheral blood T cells from recovered and acute pediatric ARDS patients. Pseudotime analysis showed preserved naive-to-memory differentiation trajectories in both CD4^+^ and CD8^+^ lineages (Figure 7A). T cell subset composition remained largely stable during pediatric ARDS recovery, with no significant changes observed in CD4^+^, CD8^+^, or γδ T cell subsets (Figures 7B–7F). Transcriptomic profiling identified the most pronounced changes in CD8^+^ effector memory and central memory T cells (Figures 7G and 7H). GO analysis showed that downregulated genes in CD8^+^ central memory T cells were enriched in granzyme-mediated cell death and lymphocyte immunity pathways (Figure 7I). Consistently, cytotoxic molecules including *SRGN*, *PRF1*, *NKG7*, *GZMB*, and *GZMH* were reduced in CD8^+^ central and effector memory T cells, whereas *GZMK* was increased in CD8^+^ effector memory T cells (Figure 7J). *CCR7* was reduced in CD4^+^ naive T cells (Figure 7J). Together, pediatric ARDS recovery is characterized by stable T cell composition but attenuated cytotoxic activation, suggesting partial immune restoration. Notably, GZMB expression in CD8^+^ effector memory T cells remained unchanged relative to both acute pediatric ARDS and pediatric controls.

**Figure 7:**
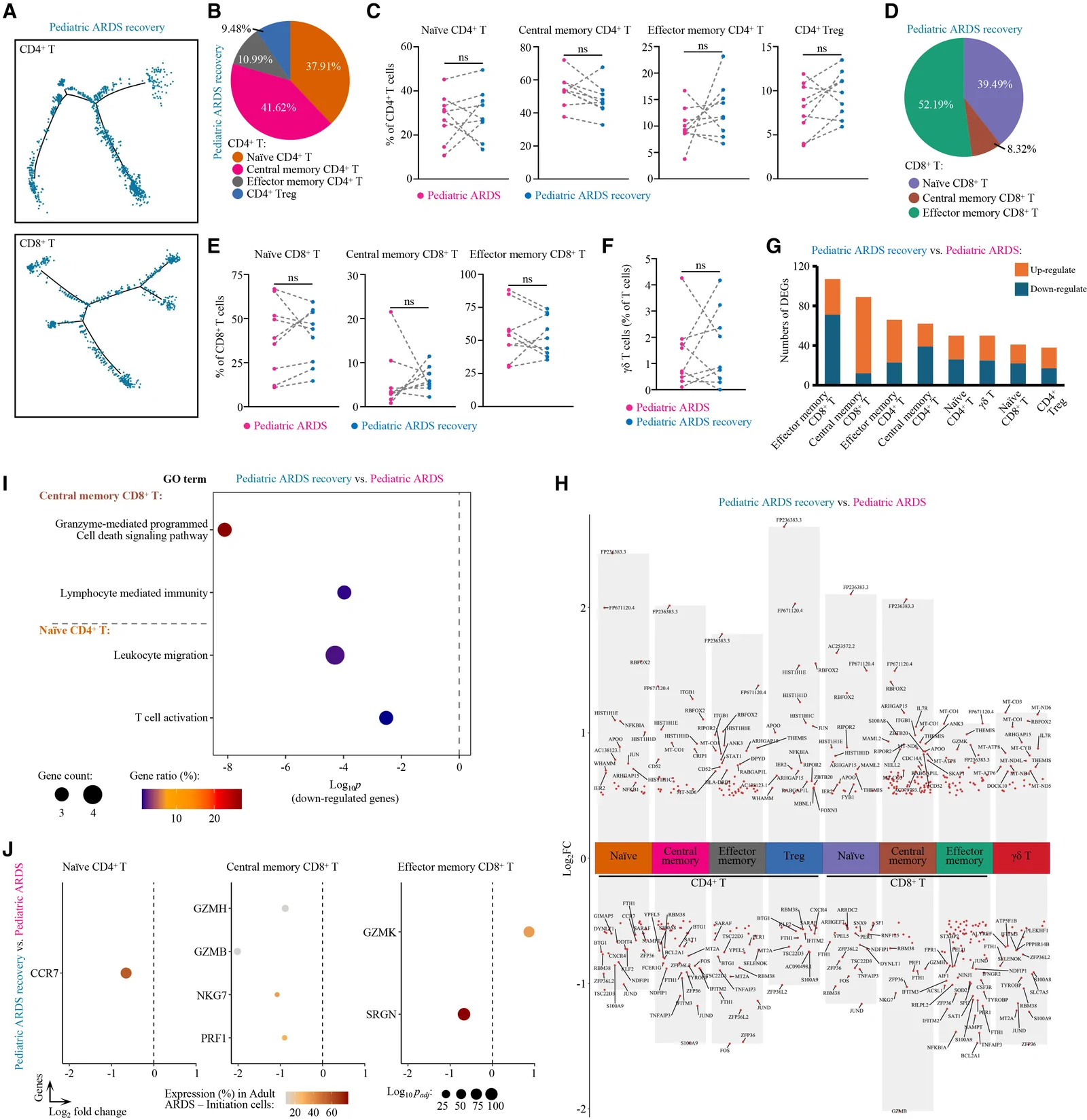
Limited changes in T cell subset composition but attenuated cytotoxic activation in pediatric ARDS recovery (A) Pseudotime trajectory analysis of CD4^+^ (top) and CD8^+^ (bottom) T cells showing preserved differentiation from naive to memory states in pediatric ARDS recovery patients. (B and C) Distribution of CD4^+^ T cell subsets (naive, central memory, effector memory, and Tregs). Pie chart (B) shows proportions within the CD4^+^ compartment. Paired plots (C) depict the percentages of CD4^+^ T cell subsets within the total CD4^+^ T cell population in paired pediatric ARDS and recovery samples. Each dot represents one sample. (D and E) Distribution of CD8^+^ T cell subsets (naive, central memory, and effector memory). Pie chart (D) shows proportions within the CD8^+^ compartment. Paired plots (E) depict the percentages of CD8^+^ T cell subsets within the total CD8^+^ T cell population in paired pediatric ARDS and recovery samples. Each dot represents one sample. (F) Paired comparison of γδ T cell abundance as a percentage of total T cells in paired pediatric ARDS and recovery samples. (G) Number of differentially expressed genes (DEGs) across subsets between pediatric ARDS recovery and pediatric ARDS. Orange indicates upregulated, and blue indicates downregulated genes. (H) Volcano plots of DEGs across subsets, showing the top 30 DEGs ranked by absolute log_2_ fold change. (I) GO enrichment of DEGs. Dot size reflects DEG counts per pathway; color denotes enrichment ratio. (J) Representative DEGs in selected subsets. Dot size represents the negative log_10_ of the adjusted *p* value, and color indicates the percentage of expressing cells. Data in (C), (E), and (F) are shown as mean ± SD. Longitudinal comparisons between pediatric ARDS and recovery samples were performed using the Wilcoxon matched-pairs signed-rank test. For comparisons involving multiple T cell subsets within each lineage, *p* values were adjusted using the Benjamini-Hochberg false discovery rate (FDR) correction. GO enrichment analysis in (I) was performed using the cumulative hypergeometric test. DEG analysis in (J) was conducted using the Wilcoxon rank-sum test with Benjamini-Hochberg correction.

### RUNX3/PRDM1-GZMB axis drives aberrant cytotoxic activation in adult ARDS

To investigate mechanisms underlying cytotoxic imbalance in adult ARDS, we analyzed upstream regulators of cytotoxic programs. Contribution analysis identified CD8^+^ effector memory T cells as the predominant source of cytotoxic granule-related genes, including *GZMB* and *PRF1* (Figures 8A and S3). Venn diagram analysis revealed consistent upregulation of *RUNX3*, *PRDM1*, and *GZMB*, together with persistent downregulation of *TCF7* in adult ARDS (Figure 8B). CD8^+^ effector memory T cells from adult ARDS exhibited increased *RUNX3*, *PRDM1*, and *GZMB* expression, accompanied by reduced *TCF7* levels (Figures 4J, 5I, 8C, and 8D).

**Figure 8:**
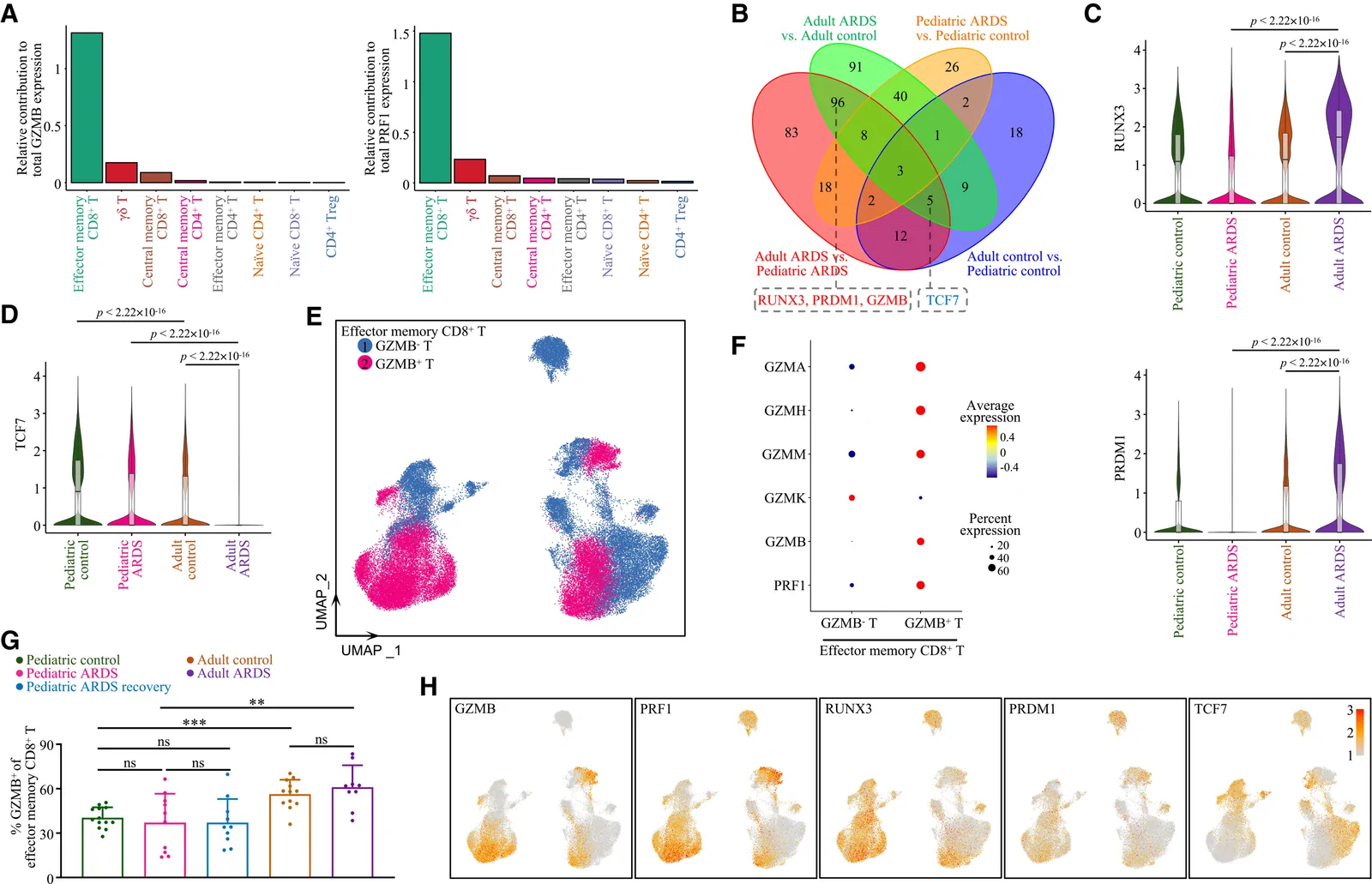
RUNX3/PRDM1 upregulation and TCF7 loss drive GZMB hyperactivation in CD8^+^ effector memory T cells (A) Bar plots showing the relative contribution of each T cell subset to total expression of *GZMB* (top) and *PRF1* (bottom). Contribution was defined as the product of mean expression per cell within each subset and the proportional abundance of that subset among total T cells. CD8^+^ effector memory T cells were the predominant contributors to both *GZMB* and *PRF1* expression. (B) Venn diagram depicting overlap of differentially expressed genes (DEGs) across four pairwise comparisons: adult ARDS vs. adult controls (green), pediatric ARDS vs. pediatric controls (orange), adult ARDS vs. pediatric ARDS (red), and adult controls vs. pediatric controls (blue). Numbers indicate DEG counts. Representative transcription factors and effector molecules are highlighted, including *RUNX3*, *PRDM1*, and *GZMB*, which were consistently upregulated in adult ARDS vs. adult controls and adult ARDS vs. pediatric ARDS, and *TCF7*, which was consistently downregulated in adult ARDS vs. adult controls, adult ARDS vs. pediatric ARDS, and adult controls vs. pediatric controls. (C and D) Violin plots showing the expression of *RUNX3*, *PRDM1* (C), and *TCF7* (D) in CD8^+^ effector memory T cells from pediatric controls, pediatric ARDS, adult controls, and adult ARDS. (E) UMAP projection showing two CD8^+^ effector memory T cell subsets: GZMB^−^, and GZMB^+^ subsets. (F) Dot plot of canonical markers (PRF1, GZMB, GZMK, GZMM, GZMH, and GZMA) confirming the molecular identities of each subset. (G) Bar plots showing the relative proportions of the GZMB^+^ subset within total CD8^+^ effector memory T cells across clinical groups. Each dot represents an individual sample. (H) UMAP visualization of subset-level distribution of *GZMB*, *PRF1*, *RUNX3*, *PRDM1*, and *TCF7* expression. Differential expression analysis in (C) and (D) was performed using the Wilcoxon rank-sum test with Benjamini-Hochberg correction. Data in (G) are presented as mean ± SD and compared using unpaired one-tailed Student’s *t* test. ∗∗*p* < 0.01, ∗∗∗*p* < 0.001.

To determine whether GZMB elevation reflected a uniform increase across CD8^+^ effector memory T cells or expansion of a distinct GZMB-high cytotoxic subset, we further reclustered CD8^+^ effector memory T cells. Based on PRF1 and granzyme family gene expression, two subsets were identified: GZMB^−^ and GZMB^+^ cells (Figures 8E and 8F). Adult controls exhibited a higher baseline frequency of GZMB^+^ cells than pediatric controls, and this age-related difference was maintained in ARDS, with selective expansion of GZMB^+^ cells in adults relative to pediatric patients (Figure 8G). Uniform manifold approximation and projection (UMAP) visualization further confirmed that GZMB^+^ cells largely lacked *TCF7* expression, consistent with transcriptional repression of this key regulatory factor (Figure 8H).^13^^,^^29^ Collectively, these findings implicate a RUNX3-PRDM1-GZMB axis, together with TCF7 downregulation, in age-dependent cytotoxic skewing in adult ARDS.

### TGFB1-mediated GZMB upregulation in CD8^+^ effector memory T cells in adult ARDS

Building on our findings of age-dependent expansion of GZMB^+^ CD8^+^ effector memory T cells in adult ARDS, we sought upstream regulators. Integrated serum proteomics revealed that TGFB1, a known inducer of RUNX3,^27^ was markedly elevated in adult ARDS compared with both adult controls and pediatric ARDS (Figures 9A and 9B and Table S4). Single-cell analysis showed increased TGFB1 expression in adult ARDS peripheral blood mononuclear cells (PBMCs) (Figure 9C). Among immune populations, neutrophils, natural killer (NK) cells, NKT cells, platelets, and T cells exhibited the largest increases, suggesting that these subsets contribute to elevated TGFB1 levels in adult patients (Figures 9D and S4A). CD8^+^ effector memory T cells expressed both TGFBR1 and TGFBR2, with broader TGFBR2 coverage, indicating their capacity to respond to TGFB1 signaling (Figures 9E and S4B).

**Figure 9:**
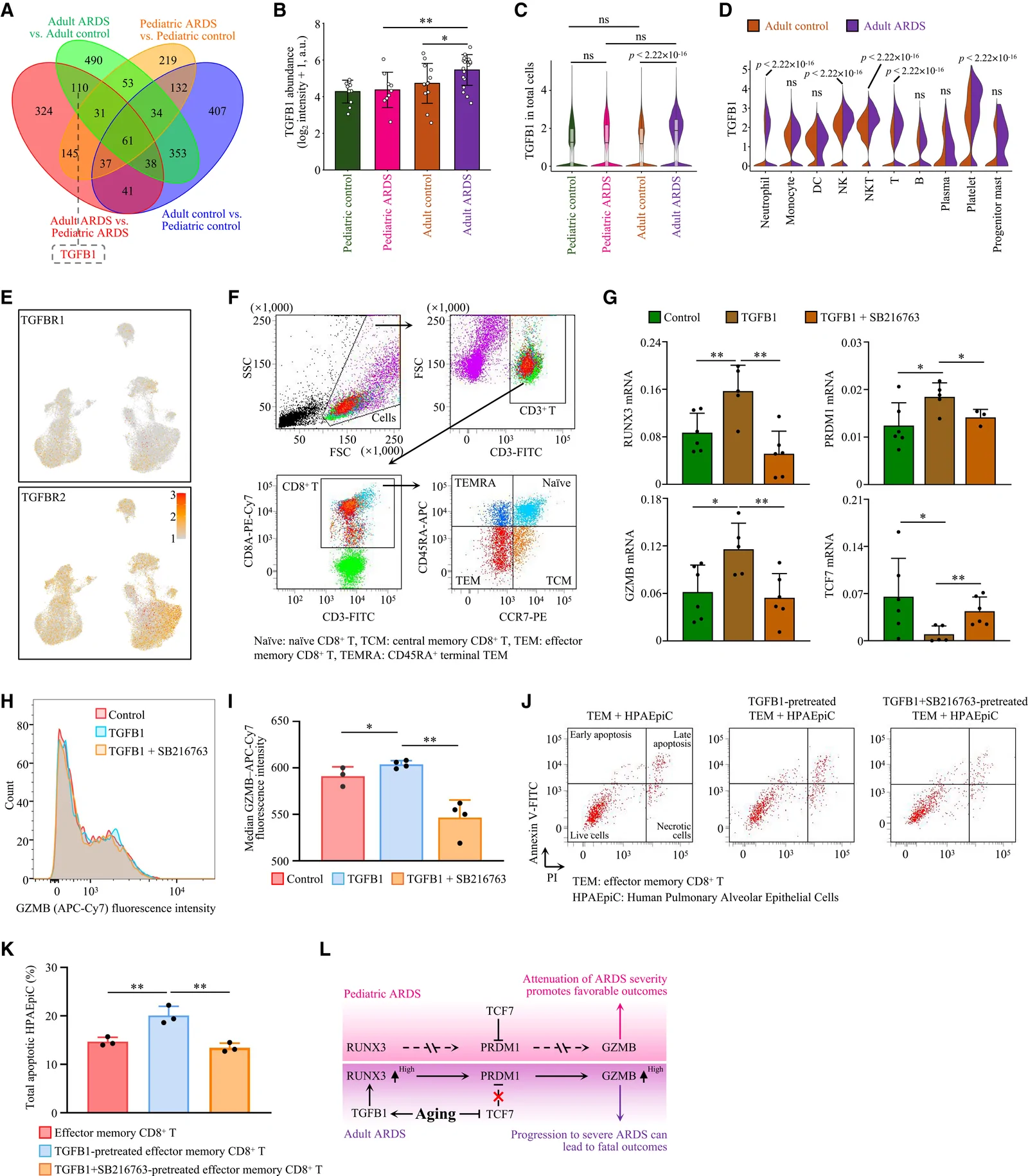
TGFB1-associated induction of GZMB^+^ CD8^+^ effector memory T cells and functional assessment of epithelial injury (A) Venn diagram showing overlap of differentially expressed proteins across serum proteomic comparisons: adult ARDS vs. adult controls (green), pediatric ARDS vs. pediatric controls (orange), adult ARDS vs. pediatric ARDS (red), and adult controls vs. pediatric controls (blue). TGFB1 was consistently upregulated in adult ARDS vs. adult controls and adult ARDS vs. pediatric ARDS. (B) Bar plot showing serum TGFB1 protein levels in pediatric controls, pediatric ARDS, adult controls, and adult ARDS. (C) Violin plot showing single-cell TGFB1 expression across all PBMCs from four clinical groups: pediatric controls, pediatric ARDS, adult controls, and adult ARDS. Each violin depicts the full distribution with overlaid boxplots indicating median and interquartile range. (D) Violin plots showing single-cell TGFB1 expression across major immune cell populations (neutrophils, monocytes, dendritic cells, NK cells, NKT cells, T cells, B cells, plasma cells, platelets, and progenitor mast cells) comparing adult controls and adult ARDS. Each violin is split by group color. (E) UMAP visualization showing the expression of TGFB1 receptors TGFBR1 (top) and TGFBR2 (bottom) across PBMCs. Each dot represents a single cell, and color intensity reflects relative expression level. (F) Flow cytometry gating and sorting strategy for CD8^+^ effector memory T cells. Representative plots illustrate sequential gating. PBMCs were first selected by forward scatter (FSC)/side scatter (SSC), then CD3^+^ T cells were identified, followed by gating on CD8^+^ subsets. CD8^+^ T cells were subsequently classified into naive, central memory (TCM), effector memory (TEM), and terminally differentiated effector memory RA^+^ (TEMRA) T cells according to CCR7 and CD45RA expression. CD8^+^ TEM cells (CCR7^−^CD45RA^−^) were finally isolated for downstream analysis. (G) Bar plots of qPCR analysis showing *RUNX3*, *PRDM1*, *GZMB*, and *TCF7* expression in CD8^+^ TEM cells under three conditions: control, TGFB1 stimulation, and TGFB1 plus SB216763 treatment. (H) Histogram overlay of GZMB protein expression (allophycocyanin-Cyanine7, APC-Cy7) in CD8^+^ effector memory T cells under the same conditions, illustrating TGFB1-induced upregulation and partial reversal by SB216763. (I) Quantification of median GZMB fluorescence intensity in CD8^+^ TEM cells. (J and K) Functional assessment of epithelial injury induced by TGFB1-conditioned CD8^+^ TEM cells. Representative annexin V/propidium iodide (PI) flow cytometry plots (J) and quantification of epithelial apoptosis (K) following co-culture of human pulmonary alveolar epithelial cells (HPAEpiCs) with control, TGFB1-treated, or TGFB1+SB216763-treated CD8^+^ TEM cells. (L) Schematic model illustrating the proposed mechanism of TGFB1-mediated regulation of GZMB in pediatric versus adult ARDS. In pediatric ARDS, relatively lower RUNX3 and PRDM1 levels, together with preserved TCF7 expression, limit GZMB induction, supporting milder disease and favorable outcomes. In adult ARDS, aging-associated increases in TGFB1 lead to upregulation of RUNX3 and PRDM1 and concomitant TCF7 downregulation, driving excessive GZMB activation, which may contribute to severe disease and poor outcomes. Data in (B), (G), (I), and (J) are presented as mean ± SD and compared using an unpaired one-tailed Student’s *t* test. ∗*p* < 0.05, ∗∗*p* < 0.01, ∗∗∗*p* < 0.001. Differential expression analysis in (C) and (D) was performed using the Wilcoxon rank-sum test with Benjamini-Hochberg correction.

For functional validation, CD8^+^ effector memory T cells were isolated from human peripheral blood^30^^,^^31^ and stimulated with TGFB1, with or without the GSK-3β inhibitor SB216763, which has been reported to enhance TCF7 expression through modulation of GSK-3β/Wnt signaling(Figure 9F).^32^^,^^33^ A dose-response experiment demonstrated that SB216763 at increasing concentrations progressively attenuated TGFB1-induced cytotoxic transcriptional remodeling, with the strongest effects observed at 5 μM (Figure S5). Therefore, 5 μM SB216763 was selected for subsequent validation experiments. TGFB1 increased RUNX3, PRDM1, and GZMB expression while suppressing TCF7; these effects were partially reversed by SB216763, as measured by qPCR (Figure 9G). Flow cytometry confirmed increased GZMB protein following TGFB1 stimulation, which was partially reduced by SB216763 (Figures 9H, 9I, and S6A). Pharmacological inhibition of TGFBR1 using SB431542 further reduced TGFB1-induced GZMB expression, supporting the involvement of TGFBR1-mediated signaling in TGFB1-driven GZMB induction (Figure S6B).

To determine whether TGFB1-induced cytotoxic remodeling of CD8^+^ effector memory T cells has functional consequences on epithelial integrity, we performed an *in vitro* co-culture assay using human CD8^+^ effector memory T cells and human pulmonary alveolar epithelial cells (HPAEpiCs). TGFB1-conditioned CD8^+^ effector memory T cells increased epithelial apoptosis, whereas SB216763 treatment attenuated this effect (Figures 9J, 9K, and S7). These findings provide functional evidence supporting a potential role of TGFB1-associated CD8^+^ T cell activation in epithelial injury. Collectively, these findings indicate that TGFB1 promotes GZMB upregulation in CD8^+^ effector memory T cells through a transcriptional program characterized by RUNX3/PRDM1 induction and TCF7 repression, potentially contributing to excessive cytotoxic activation and severe adult ARDS outcomes (Figure 9J).^27^^,^^29^^,^^34^^,^^35^^,^^36^

## Discussion

Adult patients with ARDS exhibit substantially higher mortality than pediatric patients, yet the immunological basis remains poorly understood. Importantly, our findings suggest that these differences extend beyond changes in cell frequency, reflecting a coordinated shift in CD8^+^ T cell transcriptional programs. In this study, we identified prominent features of T cell aging in adult ARDS, including reduced CD8^+^ naive T cells, contraction of the TCR repertoire, and expansion of CD8^+^ effector memory T cells. Rather than representing isolated effector gene changes, these alterations are consistent with a broader cytotoxic transcriptional program state. These cells exhibited impaired antigen recognition and homing capacity but enhanced expression of cytotoxic molecules, including GZMB and PRF1. Patient-level age-disease interaction analysis further distinguished age-associated immune remodeling from ARDS-associated transcriptional changes, revealing preferential disruption of CCR7-associated memory characteristics in adult ARDS. Single-cell transcriptomic analysis further revealed activation of the TGF-β-RUNX3-PRDM1 axis together with repression of TCF7, which was associated with excessive GZMB expression and cytotoxic imbalance in adult ARDS. These findings were supported by *in vitro* validation.

Aging is accompanied by progressive alterations in tissue and immune homeostasis.^37^ The immune system undergoes continuous remodeling and chronic low-grade inflammation, termed inflammaging, leading to progressive changes in immune cell function and composition.^38^^,^^39^^,^^40^ Among immune populations, CD8^+^ T cells are particularly sensitive to aging, showing a loss of naive cells and accumulation of effector memory subsets.^41^^,^^42^ This remodeling weakens responses to novel antigens while enhancing persistent cytotoxic activation. During acute inflammatory conditions such as ARDS, immune aging may reshape inflammatory responses and influence disease progression.^43^^,^^44^^,^^45^ These processes may converge to reshape CD8^+^ T cell transcriptional programs in a manner that promotes cytotoxic skewing during acute inflammatory stress. This framework of immune aging may help explain the higher severity and mortality observed in older individuals with infection-associated ARDS, including COVID-19.^46^^,^^47^

Our analyses identified CD8^+^ effector memory T cells as a key pathogenic subset in adult ARDS. Although GZMB normally eliminates infected cells through caspase-dependent apoptosis,^48^ excessive extracellular GZMB accumulation has been reported to degrade extracellular matrix and tight-junction proteins,^49^^,^^50^ thereby potentially damaging the alveolar epithelium and promoting tissue injury. Consistent with this concept, our co-culture experiments showed that TGFB1-conditioned CD8^+^ effector memory T cells promoted epithelial apoptosis, supporting a potential role for cytotoxic CD8^+^ T cells in bystander epithelial injury. GZMB-associated cytotoxic programs may also interact with TGF-β-associated pathways, a central mediator of fibrosis,^51^ suggesting potential involvement in inflammatory and tissue-remodeling processes during organ injury.^52^ In adult ARDS, activation of the TGF-β-RUNX3-PRDM1 signaling axis together with reduced TCF7 expression was associated with enhanced GZMB expression and cytotoxic remodeling of CD8^+^ effector memory T cells, suggesting a potential mechanism underlying age-associated immune dysregulation in ARDS. Pharmacological inhibition of TGFBR1 further supported the involvement of TGFB1 signaling in this cytotoxic reprogramming.

This program-level remodeling is also reflected in antigen receptor signaling capacity and clonal diversity. TCR repertoire analysis further demonstrated significant contraction in adult ARDS. During aging, thymic involution and diminished clonal renewal capacity^53^^,^^54^ progressively reduce TCR diversity, thereby narrowing antigen recognition breadth and constraining immune responses to novel pathogens.^13^^,^^55^ In addition, we observed a coordinated downregulation of TCR complex components (CD3D, CD3E, and CD3G) together with the co-receptor CD8B, suggesting impaired signal transduction and amplification following antigen recognition in CD8^+^ T cells of adult ARDS patients. The coexistence of diminished antigen-specific recognition and GZMB-driven bystander killing may exacerbate pulmonary injury through both excessive nonspecific cytotoxicity and insufficient antigen-specific immune responses, thereby providing a molecular basis for immune imbalance and tissue injury in adult ARDS.

Collectively, this study provides a new immunological framework for understanding the divergent outcomes of adult and pediatric ARDS, in which adult ARDS represents a state of immune maladaptation characterized by excessive cytotoxic activation and limited antigen-specific response. These findings suggest potential precision immunomodulatory strategies, including modulating the TGF-β-RUNX3-PRDM1-GZMB/TCF7 axis, targeting granzyme-mediated bystander injury, and preserving TCR diversity and the naive T cell pool to restore immune homeostasis.

### Limitations of the study

This study has several limitations. First, although inclusion of a pediatric ARDS post-treatment cohort allowed us to evaluate immune remodeling after clinical improvement, a corresponding adult recovery cohort was not available. The higher mortality of severe adult ARDS patients requiring invasive mechanical ventilation or extracorporeal membrane oxygenation (ECMO) limited the number of survivors eligible for longitudinal follow-up and recovery-phase sampling. Therefore, whether the cytotoxic reprogramming observed in adult ARDS is reversible or persists after recovery remains unclear. Future longitudinal studies incorporating age-matched adult and pediatric ARDS recovery cohorts are needed to define age-dependent immune resolution trajectories. Second, as this work primarily relied on single-cell analysis of peripheral blood, it may not fully reflect the dynamic immune microenvironment within the lung, which is critical to ARDS pathogenesis. Airway samples and epithelial injury biomarkers were unavailable in this cohort, preventing direct assessment of pulmonary cytotoxic activity in patients. Although our co-culture experiments provide functional evidence that TGFB1-conditioned CD8^+^ effector memory T cells can promote epithelial apoptosis, whether this cytotoxic remodeling also affects antiviral clearance capacity remains unclear and requires further investigation using influenza infection models. Future studies integrating bronchoalveolar lavage fluid profiling, spatial analyses, and circulating immune signatures will be required to further establish the spatial relationship between cytotoxic T cell activation and lung injury. Moreover, the RUNX3-PRDM1-GZMB axis identified in peripheral CD8^+^ effector memory T cells requires further investigation to determine how systemic immune remodeling corresponds to immune alterations within lung tissue. Although pharmacological inhibition of TGFBR1 supported the involvement of TGFB1 signaling in this cytotoxic remodeling program, direct genetic approaches targeting RUNX3 will be required to establish its causal role in linking TGFB1 signaling to PRDM1 induction and TCF7 repression. Furthermore, SB216763 primarily functions as a GSK-3β inhibitor rather than a selective TCF7 activator and may influence additional downstream signaling pathways beyond TCF7 regulation. Therefore, future studies using genetic approaches targeting TCF7 will be required to establish its direct causal role in CD8^+^ T cell cytotoxic reprogramming.

In conclusion, this study systematically delineates the functional differences of T cells between adult and pediatric ARDS and identifies a RUNX3-PRDM1-GZMB-driven mechanism underlying aberrant cytotoxic activation. These findings provide new mechanistic insight into the immunopathology of adult ARDS and suggest potential therapeutic targets for developing T cell-based precision interventions. Collectively, these findings support a model in which adult ARDS is characterized by a TGFB1-associated shift in CD8^+^ T cell transcriptional programs toward a TCF7-low cytotoxic state-like configuration.

## Resource availability

### Lead contact

Requests for further information and resources should be directed to and will be fulfilled by the lead contact, Huaiyong Chen (huaiyong.chen@foxmail.com).

### Materials availability

This study did not generate new, unique reagents.

### Data and code availability

• The raw single-cell RNA sequencing and T cell receptor sequencing data generated in this study have been deposited in the Genome Sequence Archive for Human (GSA-Human; https://ngdc.cncb.ac.cn/gsa-human/) at the National Genomics Data Center, China National Center for Bioinformation/Beijing Institute of Genomics, Chinese Academy of Sciences, under accession number GSA-Human: HRA021060. Access to raw human sequencing data is controlled and subject to approval in accordance with the ethical approvals, informed consent, and institutional data governance policies of the Chinese PLA General Hospital. Requests to access or reuse sequencing data, clinical metadata, or other controlled-access datasets generated from participants must be reviewed and approved by Lixin Xie (xielx301@126.com). Source data generated in this study are provided with this paper where permitted.
• Analysis code supporting the findings of this study is available from the lead contact upon reasonable request and subject to institutional approval.
• Other non-identifiable data supporting the findings of this study are available from the lead contact upon reasonable request and subject to institutional approval.

## Acknowledgments

This work was supported by the Ministry of Science and Technology of the People’s Republic of China (grant no. 2024YFA1108906) and Tianjin Key Medical Discipline Construction Project (grant no. TJYXZDXK-3-018B). We acknowledge the clinical teams at the Seventh and Eighth Medical Centers of the 10.13039/501100009580Chinese PLA General Hospital for cohort recruitment, clinical sample collection, clinical data management, and support for multi-omics data generation. The figures were generated using BioRender.com.

## Author contributions

L.X. and H.C. conceptualized the study, supervised the project, and provided overall scientific direction. L.X. led the clinical study design, coordinated the clinical cohort at the Chinese PLA General Hospital, supervised clinical sample acquisition and data governance, and oversaw ethical compliance and clinical interpretation. K.L., K.A., and L.S. performed single-cell and TCR repertoire analyses. L.S. was responsible for the single-cell RNA-seq and TCR-seq workflow, proteomic sequencing workflow coordination, sequencing quality control, adult control and adult ARDS sample collection, clinical indicator interpretation, and sequencing data management. X.H. was responsible for pediatric control and pediatric ARDS clinical sample collection, pediatric clinical data acquisition, and clinical data management. J.H. and Y.Y. contributed to the interpretation of clinical data. K.L., K.A., Y.L., H.L., S.X., Y.W., J.Y., and L.L. performed experimental validations. K.L., K.A., and H.C. drafted the manuscript. X.W., X.H., L.X., and H.C. critically reviewed and revised the manuscript. All authors approved the final manuscript.

## Declaration of interests

The authors declare no competing interests.

## STAR★Methods

### Key resources table

**Table:** 

REAGENT or RESOURCE	SOURCE	IDENTIFIER
**Antibodies**		

PE/Cyanine7 anti-human CD8a	Biolegend	Cat#: 301012; RRID: AB_314130
APC anti-human CD45RA	Biolegend	Cat#: 304111; RRID: AB_314415
PE anti-human CD197(CCR7)	Biolegend	Cat#: 353203; RRID: AB_10916391
FITC anti-human CD3	Biolegend	Cat#: 344803; RRID: AB_2043992
APC/Cyanine7 anti-human/mouse Granzyme B Recombinant Antibody	Biolegend	Cat#: 372227; RRID: AB_2936711
APC/Cyanine7 anti-human CD326 (Ep-CAM)	Biolegend	Cat#: 324245; RRID: AB_2783193
FITC Annexin V Apoptosis Detection Kit with PI	Biolegend	Cat#: 640914

**Biological samples**		

Peripheral blood samples	Tianjin Haihe Hospital	Approval No. 2025HHKT-025

**Chemicals, peptides, and recombinant proteins**		

RPMI 1640 Medium	Cellgro	Cat#: 10-040-CVRC
Fetal Bovine Serum(Defined)	Excell	Cat#: FSD500
Penicillin-streptomycin	Gibco	Cat#: 15140-122
eBioscience™ 1× RBC lysis buffer	Invitrogen	Cat#: 00-4333-57
Histopaque®-1077	Sigma-Aldrich	Cat#: 10771
0.25% Trypsin-EDTA	Gibco	Cat#: 25200-072
TRIzol reagent	Invitrogen	Cat#: 15596018
HiScript III RT SuperMix for qPCR (+gDNA wiper)	Vazyme	Cat#: R323-01
ChamQ Universal SYBR qPCR Master Mix	Vazyme	Cat#: Q711-03
TGFB1 Protein, Human (HEK293)	MCE	Cat#: HY-P70543
SB216763	Sigma-Aldrich	Cat#: S3442
SB431542	Sigma-Aldrich	Cat#: S4317
Complete Culture Medium for Immortalized Human Type II Alveolar Epithelial Cells	Shanghai Jinyuan Biotechnology Co., Ltd.	Cat#: JY-Y842

**Critical commercial assays**		

eBioscienceTM Foxp3/Transcription Factor Fixation/Permeabilization Kit	ThermoFisher	Cat#: 00-5523-00
10× Genomics 5′ Library Construction Kit v2	10× Genomics	Cat#: PN-1000263

**Deposited data**		

Single-cell RNA sequencing and T cell receptor sequencing data	Genome Sequence Archive for Human (GSA-Human), National Genomics Data Center, China National Center for Bioinformation/Beijing Institute of Genomics, Chinese Academy of Sciences	Accession code: HRA021060

**Experimental models: Cell lines**		

HPAEPiC	Shanghai Jinyuan Biotechnology Co., Ltd.	Cat#: JY-J002

**Oligonucleotides**		

Human-β-actin Fwd: CACTCTTCCAGCCTTCCTTC	Sangon Biotech Co., Ltd.	N/A
Human-β-actin Rev: GTACAGGTCTTTGCGGATGT	Sangon Biotech Co., Ltd.	N/A
Human-GZMB Fwd: ACACTCACACACACTACAAGAG	Sangon Biotech Co., Ltd.	N/A
Human-GZMB Rev: ACGCACAACTCAATGGTACT	Sangon Biotech Co., Ltd.	N/A
Human-RUNX3 Fwd: GCAGGCAATGACGAGAACTA	Sangon Biotech Co., Ltd.	N/A
Human-RUNX3 Rev: CAGTGATGGTCAGGGTGAAA	Sangon Biotech Co., Ltd.	N/A
Human-PRDMI Fwd: CAGGTCTGCCACAAGAGATTTA	Sangon Biotech Co., Ltd.	N/A
Human-PRDMI Rev: CACCTTGCATTGGTATGGTTTC	Sangon Biotech Co., Ltd.	N/A
Human-TCF7 Fwd: GGACAACTACGGGAAGAAGAAG	Sangon Biotech Co., Ltd.	N/A
Human-TCF7 Rev: TGGGCTAGAGGAAGAAGACA	Sangon Biotech Co., Ltd.	N/A

**Software and algorithms**		

Seurat package	Satija Lab	v4.3.0
R	Satija Lab	v4.3.1
Cell Ranger	Satija Lab	v4.0.0
GraphPad Prism 10	GraphPad Software	v10.4.1
PrimerQuest Tool	Integrated DNA Technologies	https://www.idtdna.com/primerquest/home/results
FlowJo™ Software	FlowJo LLC	v10.8.1

**Other**		

EDTA-anticoagulated Vacutainer tubes	BD	Cat#: 367861

### Experimental model and study participant details

#### Sex as a biological variable

This study included both male and female human participants. Sex was recorded as part of the clinical metadata but was not considered the primary biological variable of interest, as the study was designed to investigate age-associated immune differences in ARDS. Because of the limited sample size within each disease and age group, the study was not powered to detect sex-specific effects.

#### Patient recruitment and sample collection

A total of 67 peripheral blood samples were obtained from 58 participants between June 2022 and September 2024 at the Seventh and Eighth Medical Centers of the Chinese PLA General Hospital, including pediatric and adult intensive care units (ICUs) and the health examination center. The study protocol was approved by the Ethics Committee of the PLA General Hospital (approval No. 2022052701006), and written informed consent was obtained from all participants or their legal guardians. Inclusion criteria were ICU admission with a confirmed diagnosis of severe ARDS secondary to viral pneumonia, evidence of recent influenza virus infection, and age ≤15 years (pediatric) or ≥45 years (adult). Recent influenza virus infection was defined by serum IgM positivity against influenza A virus or influenza B virus, detected using an indirect immunofluorescence assay with a commercial kit (Vircell, S.L., Granada, Spain) in serum isolated from peripheral venous blood collected within 1 h after admission; the assay was performed according to the manufacturer’s instructions with internal positive and negative controls. Exclusion criteria included respiratory failure caused by other acute or chronic pulmonary diseases, withdrawal of resuscitation or terminal condition, and severe comorbidities such as immunosuppression, prior organ transplantation, or disseminated malignancy. All patients received standardized critical care management.

Among these samples, 54 peripheral blood samples from 45 participants were used for single-cell RNA-seq and TCR-seq. Participants were stratified by age and disease status. The pediatric control group consisted of 13 children undergoing elective surgery without infection or acute inflammation, and the adult control group included 12 healthy adults recruited from the health examination center. Eleven pediatric patients were diagnosed with ARDS; among them, 10 blood samples collected within 24 h after initiation of invasive mechanical ventilation (IMV) or extracorporeal membrane oxygenation (ECMO) were assigned to the pediatric ARDS group, and another 10 post-treatment samples collected after recovery and successful withdrawal of life support were classified as the pediatric ARDS post-treatment group. For non-survivors, a terminal sample was obtained immediately before discontinuation of life support. The adult ARDS group included nine patients, and all samples were collected only within 24 h after IMV or ECMO initiation. ARDS diagnosis was established according to the Berlin definition and the Pediatric Acute Lung Injury Consensus Conference (PALICC) criteria.^56^^,^^57^ Samples used for routine blood testing and serum proteomic analysis were grouped according to the same age- and disease-status framework as the single-cell RNA-seq/TCR-seq cohort. Clinical characteristics, demographic information, and viral testing results are summarized in Tables S1, S2, S3, and S4.

### Method details

#### Peripheral blood processing for scRNA-seq

For each participant, 3 mL of venous blood was collected into EDTA-anticoagulated Vacutainer tubes (BD, Cat# 367861) and gently inverted to mix. Samples were centrifuged at 500 g for 5 min at room temperature, and the plasma supernatant was removed. Approximately 1 mL of the remaining blood cells was diluted 1:1 with PBS containing 1% fetal bovine serum (FBS; ExCell, Cat# FSD500). The mixture was gently layered over 2 mL of Histopaque-1077 (Sigma-Aldrich, Cat# 10771) and centrifuged at 700 g for 20 min at room temperature (with brake off). Peripheral blood mononuclear cells (PBMCs) were collected from the plasma–Ficoll interface. The lower red pellet (granulocyte/erythrocyte layer) was aspirated (1 mL) and treated with 10 mL of red blood cell lysis buffer (invitrogen, Cat# 00–4333-57) for 10 min, followed by centrifugation at 300*g* to obtain granulocytes. After counting, PBMCs and granulocytes were mixed at a 2:1 ratio and adjusted to a final concentration of 7–12 × 10^5^ cells/mL with viability >80%. Single-cell libraries were prepared using the 10× Genomics 5′ Library Construction Kit v2 (10× Genomics, Cat# PN-1000263).

#### Single-cell RNA-seq data processing and integration

Raw sequencing data were processed using Cell Ranger (v4.0.0) with the GRCh38 human reference genome to generate gene–cell expression matrices. All samples were analyzed in R (v4.3.1) using the Seurat package (v4.3.0). Quality control was performed based on UMI counts and gene features, retaining cells with 200–5,000 detected genes and <10% mitochondrial transcripts. Data were then normalized, highly variable genes (top 2,000) were identified, and scaling was performed while regressing out mitochondrial transcript percentage. Principal component analysis (PCA) was applied for dimensionality reduction and identification of major sources of variation. Batch and inter-individual effects were corrected using the Harmony algorithm (v0.1.1) at the sample level. The top 30 principal components determined by the elbow criterion were used to construct nearest-neighbor graphs for graph-based clustering (resolution = 1.5) and visualization via UMAP embedding. Doublets were identified and removed using DoubletFinder (v2.0.3), where the expected doublet rate was estimated according to the 10× Genomics multiplet rate and sample-specific cell recovery, adjusted by homotypic proportion modeling, and only cells annotated as “Singlet” were retained for downstream analyses. The integrated single-cell dataset was subsequently used for identification of major immune cell subsets, differential gene expression analysis, pathway activity scoring, and other downstream analyses. Cell-type identities were assigned according to canonical marker gene expression and manually curated with reference to established immune cell profiles.

#### Differential expression and data visualization

Differential expression analysis was performed using the FindMarkers function in Seurat (Wilcoxon rank-sum test, default parameters) for pairwise group comparisons. DEGs were defined by thresholds of min.pct = 0.25 and logfc.threshold = 0.5, with significance determined at Bonferroni-adjusted *p* values (p_val_adj <0.05). All significant DEGs were used for downstream visualization and statistical analyses. Heatmaps were generated with ComplexHeatmap (v2.16.0). Target genes were ordered by cell subset and effect direction, and normalized expression values (slot = “data”) were extracted and averaged across each “sample–group–cell type” combination. Gene-wise *Z* score normalization was then applied, and a continuous gradient (colorRamp2(-2, 0, 2)) was used for visualization. Violin plots were generated using VlnPlot. To enhance readability, data points were hidden (pt.size = 0), and boxplots (geom_boxplot, outlier.shape = NA) were overlaid to depict expression distributions. Multi-comparison volcano plots were generated in R using tidyverse, ggplot2, and ggrepel. The input was an integrated DEG table containing avg_log2FC, PValue (adjusted *p* value, p_val_adj), Cluster (cell subset name), and Name (gene symbol). Significance was defined as adjusted *p* < 0.05 with |log2FC| ≥ 0.5. For each Cluster, the top genes ranked by absolute log_2_FC (default *n* = 30) were labeled with ggrepel to highlight key DEGs. Dot plots were generated using ggplot2, with the x axis representing log_2_FC, dot size corresponding to −log10(*p* value), and color indicating the proportion of cells expressing each gene across groups. A small constant (1 × 10^−300^) was added to avoid logarithmic overflow.

#### Age–disease interaction analysis and patient-level validation

CD8^+^ effector memory T cells were extracted from the single-cell RNA-sequencing dataset according to cell-type annotation. Raw count matrices were aggregated at the patient level using the AggregateExpression function in Seurat. Each patient was considered as an independent biological replicate to avoid potential inflation of statistical significance resulting from treating individual cells as independent observations. Differential expression analysis was performed using DESeq2 with the design formula:

Expression∼Age+Disease+Age:Disease

The coefficient of the age × disease interaction term (AgeAdult:DiseaseARDS) was extracted to identify transcriptional changes associated with differential responses to ARDS between adults and children. Genes with adjusted *p* value < 0.05 and absolute log2 fold change >0.5 were considered significant interaction-associated genes.

For patient-level validation, CCR7 expression was calculated by averaging CCR7 expression across CD8^+^ effector memory T cells from each patient. A TCF7-associated memory module score was calculated using Seurat AddModuleScore based on TCF7, CCR7, IL7R, SELL, LTB, and MAL, followed by averaging scores across cells from each patient. The age–disease interaction of the memory module score was evaluated using a linear regression model including age, disease status, and the age–disease interaction term, and the interaction coefficient and corresponding *p* value were extracted.

#### Pathway activity scoring

Pathway activity scores were calculated using the AddModuleScore function in Seurat. Gene sets were obtained from the GSEA Molecular Signatures Database (MSigDB; https://www.gsea-msigdb.org/gsea/index.jsp) and included four biological pathways: GOBP_CYTOKINE_MEDIATED_SIGNALING_PATHWAY.v2025.1.Hs, GOBP_GRANZYME_MEDIATED_PROGRAMMED_CELL_DEATH_SIGNALING_PATHWAY.v2025.1.Hs, GOBP_LYMPHOCYTE_MIGRATION_INTO_LYMPHOID_ORGANS.v2025.1.Hs, and GOCC_T_CELL_RECEPTOR_COMPLEX.v2025.1.Hs. The complete gene lists included in each module are provided in Table S5. For each cell, the AddModuleScore algorithm calculates module activity by subtracting the average expression of randomly selected control genes matched for average expression level from the average expression of genes within the predefined gene set:

Modulescore=Mean(targetgenesetexpression)−Mean(controlgenesetexpression)

Module score comparisons between groups were performed using Wilcoxon rank-sum tests. For analyses involving multiple T cell subsets or multiple group comparisons, *p* values were adjusted using the Benjamini–Hochberg false discovery rate (FDR) correction for multiple comparisons. Adjusted *p* values (FDR) < 0.05 were considered statistically significant.

#### TCR repertoire and clonotype analysis

The 10× Genomics V(D)J sequencing data were imported by sample and integrated using the combineTCR function in scRepertoire (v1.10.1). The proportion of strictly paired clonotypes was quantified using quantContig (cloneCall = “strict”, scale = TRUE), representing the relative abundance of TRA+TRB paired clonotypes per sample. Results were visualized as bar plots (mean ± SD) overlaid with individual data points. Group comparisons were performed using the Wilcoxon rank-sum test, with statistical significance defined as *p* < 0.05. Clonal abundance was categorized with clonalHomeostasis (cloneCall = “strict”) into predefined frequency classes: Rare (0 < X ≤ 1 × 10^−4^), Small (≤1 × 10^−3^), Medium (≤1 × 10^−2^), Large (≤1 × 10^−1^), and Hyperexpanded (≤1). Clonal diversity was estimated for each sample using clonalDiversity, primarily reporting the Shannon and Inverse Simpson indices (cloneCall = “gene”, bootstrap resampling n.boots = 100). All visualizations and statistical annotations were generated using ggplot2 and ggpubr, with color schemes and group order specified in the corresponding figure legends.

#### Pseudotime trajectory inference

Pseudotime trajectory analyses were performed for CD4^+^ T, CD8^+^ T, and CD8^+^ effector memory T subsets using Monocle (v2.28.0) with identical analytical settings. Single-cell transcriptomic data for each subset were extracted from Seurat objects corresponding to samples across different clinical groups of the study. The raw UMI count matrix and corresponding metadata were converted into CellDataSet objects using newCellDataSet() with a negative binomial model (expressionFamily = negbinomial.size()). Normalization and dispersion estimation were performed with estimateSizeFactors() and estimateDispersions(), retaining genes expressed in at least five cells for downstream analysis. DEGs were identified using differentialGeneTest(), and those with q < 0.01 were used as ordering genes. Dimensional reduction and trajectory inference were performed using reduceDimension() (method = “DDRTree”), followed by orderCells() to assign pseudotime. The root state was defined based on biologically naive T cells to indicate the direction of differentiation. Cell trajectories were visualized using plot_cell_trajectory(), and all figures were refined in ggplot2 for consistent color schemes and aesthetics across subsets.

#### Gene ontology enrichment and visualization

GO enrichment analysis was performed using the Metascape web platform (https://metascape.org/gp/index.html). For each comparison, upregulated and downregulated DEGs were analyzed separately, with the organism set to Homo sapiens and other parameters kept at default. Enrichment significance was assessed using a cumulative hypergeometric test, and terms with *p* < 0.01 were considered significantly enriched. The enrichment results were exported and visualized in R. Visualization was conducted using ggplot2. For upregulated genes, enrichment significance was plotted as −log_10_(*p* value), while for downregulated genes it was plotted as log10 (*p* value). GO terms were displayed on the y axis, and significance values on the x axis. Dot size represented the number of genes enriched in each term (Count), and dot color reflected the gene ratio (GeneRatio, as provided by Metascape).

#### Relative contribution of T cell subsets to gene expression

To quantify the relative contribution of different T cell subsets to the overall expression of key cytotoxic molecules (*GZMB*, *PRF1*, *GZMA*, *GZMH*, *GZMK*, *NKG7*), calculations were performed using the Seurat workflow. Normalized single-cell RNA-seq expression matrices were extracted and converted to a linear scale to ensure accuracy of subsequent weighted computations. For each subset, the mean expression level was multiplied by its proportional abundance within total T cells to obtain a contribution score (mean expression × subset proportion). This score reflects the weighted contribution of each subset to the total expression of the target gene. Visualization was performed using ggplot2.

#### Serum proteomic analysis

Serum samples were depleted of high-abundance proteins using High-Select™ Top14 Abundant Protein Depletion Mini Spin Columns (Thermo Fisher Scientific, USA), digested with trypsin (Promega, USA) at 37°C for 16 h, and desalted. Peptides were separated on an EASY-nLC 1200 liquid chromatography system (Thermo Fisher Scientific, USA) and analyzed on an Orbitrap Exploris 480 mass spectrometer (Thermo Fisher Scientific, USA) operated in data-independent acquisition (DIA) mode (m/z 300–1400, resolution 60,000 at m/z 200). According to the serum proteomic results, statistical analyses were performed among different sample groups.

#### Isolation of peripheral blood mononuclear cells and sorting of CD8^+^ effector memory T cells

Peripheral blood samples were collected from healthy donors (aged 20–45 years, both sexes) at Tianjin Haihe Hospital Health Examination Center. All participants provided written informed consent, and the study was approved by the Ethics Committee of Tianjin Haihe Hospital (Approval No. 2025HHKT-025). Blood samples were collected using EDTA as an anticoagulant. PBMCs were isolated by Ficoll density-gradient centrifugation, followed by washing with PBS containing 1% FBS and red blood cell lysis. CD3^+^CD8^+^CCR7^−^CD45RA^−^ effector memory T cells were purified by flow cytometric sorting using the following antibodies: CD3-FITC (BioLegend, Cat# 344803), CD8a-PE/Cy7 (BioLegend, Cat# 301012), CCR7-PE (BioLegend, Cat# 353203), and CD45RA-APC (BioLegend, Cat# 304111).

Sorted CD8^+^ effector memory T cells were plated at 5×10^4^ cells/well in RPMI 1640 supplemented with 10% FBS. Cells were assigned to three groups: control, TGFB1 stimulation (3 ng/mL; MCE, Cat# HY-P70543), and TGFB1 (3 ng/mL) plus GSK-3β inhibitor SB216763 (5 μM; MCE, Cat# S3442). A preliminary dose-response experiment was performed by co-treating CD8^+^ effector memory T cells with TGFB1 and SB216763 at concentrations of 1, 2.5, or 5 μM. The effects of different concentrations were evaluated by measuring TCF7, RUNX3, and GZMB expression using quantitative PCR. Based on the concentration-dependent regulation of these transcriptional markers, 5 μM SB216763 was selected for subsequent validation experiments. To further assess the involvement of TGFBR1-mediated signaling in TGFB1-induced cytotoxic activation, cells were pretreated with the TGFBR1 inhibitor SB431542 (5 μM; Sigma-Aldrich, Cat# S4317) for 1 h and subsequently stimulated with TGFB1 (3 ng/mL) in the continued presence of SB431542 for 6 h. The concentration of SB431542 was selected based on previous studies.^58^^,^^59^ Following stimulation, cells were collected, washed with 1 mL PBS containing 1% FBS, and fixed/permeabilized using the eBioscience™ Foxp3/Transcription Factor Fixation/Permeabilization Kit (ThermoFisher, Cat# 00–5523-00). Intracellular staining was performed with directly conjugated antibody Granzyme B-APC/Cy7 (BioLegend, Cat# 372227). Stained cells were washed and resuspended in PBS containing 1% FBS for flow cytometry acquisition. For RNA analysis, cells were harvested and processed for RNA isolation and qPCR under the same stimulation conditions.

#### CD8^+^ effector memory T–HPAEpiC co-culture assay

To evaluate the functional consequences of TGFB1-induced CD8^+^ effector memory T remodeling on epithelial injury, sorted human CD8^+^ effector memory T cells (CCR7^−^CD45RA^−^) were stimulated with control medium, TGFB1 (3 ng/mL), or TGFB1 plus SB216763 (5 μM) for 6 h under the conditions described above. Human pulmonary alveolar epithelial cells (HPAEpiCs; Shanghai Jinyuan Biotechnology Co., Ltd., Cat# JY-J002) were cultured according to the manufacturer’s instructions. Subsequently, treated CD8^+^ effector memory T cells were co-cultured with HPAEpiCs for 24 h. After co-culture, HPAEpiCs were identified using APC/Cy7 anti-human EpCAM antibody (BioLegend, Cat# 324245), and epithelial apoptosis was assessed using the FITC Annexin V Apoptosis Detection Kit with PI (BioLegend, Cat# 640914) according to the manufacturer’s instructions. Representative flow cytometry gating strategy is shown in Figure S7.

### Quantification and statistical analysis

Comparisons of blood cell counts, cellular proportions, qPCR gene expression data between two groups were performed using unpaired Student’s *t*-tests, with statistical significance defined as *p* < 0.05. Single-cell transcriptomic and TCR-related differential analyses were conducted in the R environment, and GO enrichment was performed using the Metascape online platform. For longitudinal comparisons between pediatric ARDS and recovery samples, only patients with matched acute and recovery samples were included. Paired comparisons were performed using the Wilcoxon matched-pairs signed-rank test, with each patient considered as an individual matched pair. For comparisons involving multiple T cell subsets within the each lineage, *p* values were adjusted using the Benjamini–Hochberg false discovery rate correction. Detailed analytical and statistical procedures are described in the corresponding sections above.

## Supplemental information

Document S1. Figures S1–S7

Table S1. Baseline characteristics of the single-cell sequencing cohort

Table S2. Individual-level clinical and omics metadata for all peripheral blood samples

Table S3. Baseline characteristics of the peripheral blood examination cohort

Table S4. Baseline characteristics of the serum proteomics cohort

Table S5. MSigDB module gene sets
