# Supplementary material for: CD8+ T cell transcriptional reprogramming toward TCF7-low cytotoxicity drives immune aging in viral ARDS
Source: iScience. 2026 Oct 8;29(11):117774. doi: 10.1016/j.isci.2026.117774 (PMC13676981; doi:10.1016/j.isci.2026.117774)
Supplement: Supplementary file 1 [file mmc1.pdf]

iScience, Volume *29*

Supplemental information

CD8^{+} T cell transcriptional reprogramming toward TCF7-low cytotoxicity drives immune aging in viral ARDS

Kuan Li, Kaibinuer Ainiwaer, Licheng Song, Jinmiao Hu, Yafan Yang, Yimeng Liu, Hongwei Li, Shiqi Xu, Youyang Wang, Juan Yin, Li Li, Xing Wang, Xiaoyang Hong, Lixin Xie, and Huaiyong Chen

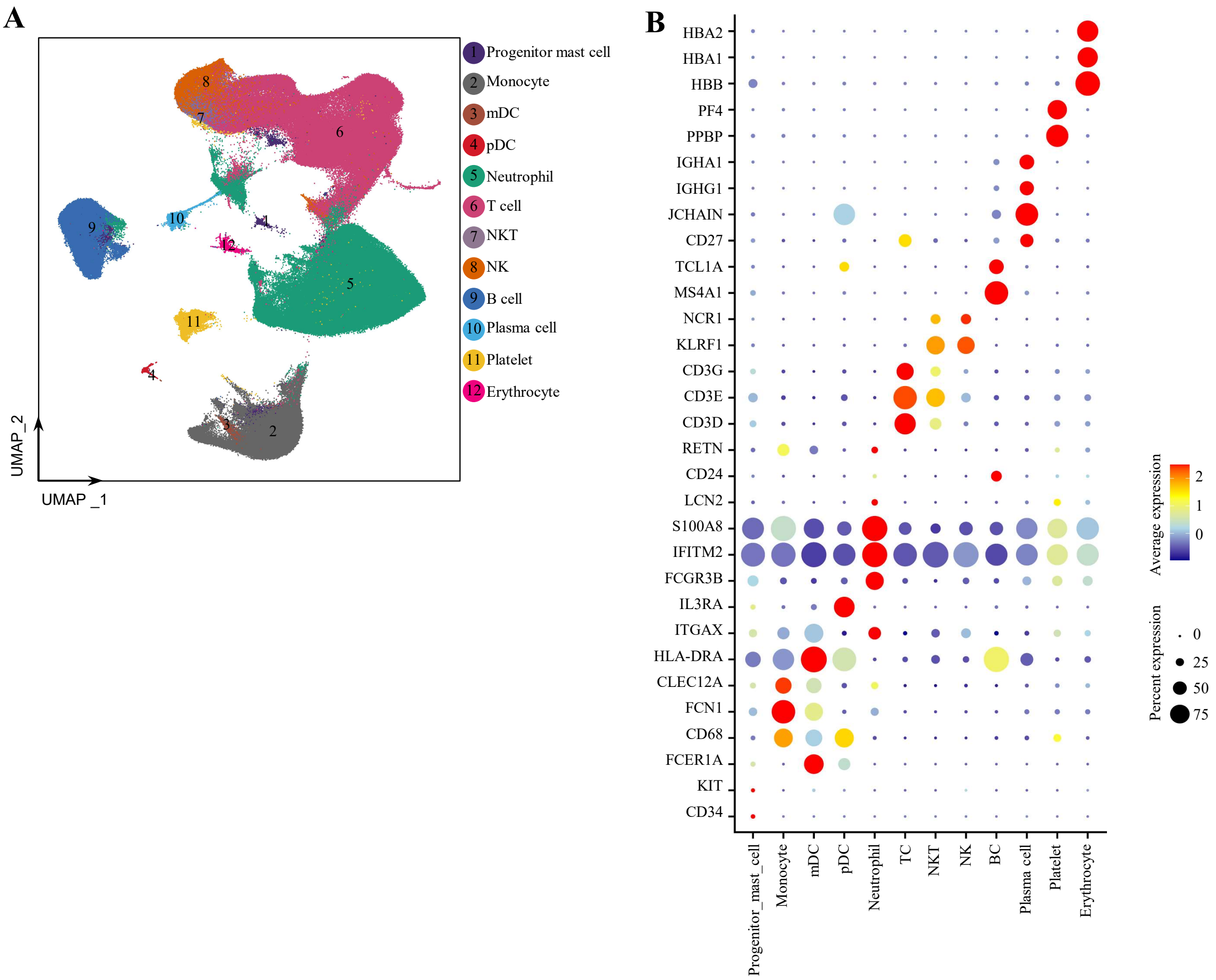

Figure S1. Single-cell transcriptomic atlas of human peripheral blood immune cells.

A. UMAP visualization of single-cell RNA sequencing data from human peripheral blood, showing clustering of major immune cell populations. Each color represents a distinct cell type: progenitor mast cell, monocyte, myeloid dendritic cell (mDC), plasmacytoid dendritic cell (pDC), neutrophil, T cell, NKT, NK, B cell, Plasma cell, Platelet, and Erythrocyte.

B. Dot plot showing expression of canonical marker genes across identified cell types. Dot size represents the percentage of cells expressing each gene within a cluster, while color intensity indicates the average expression level (log-normalized).

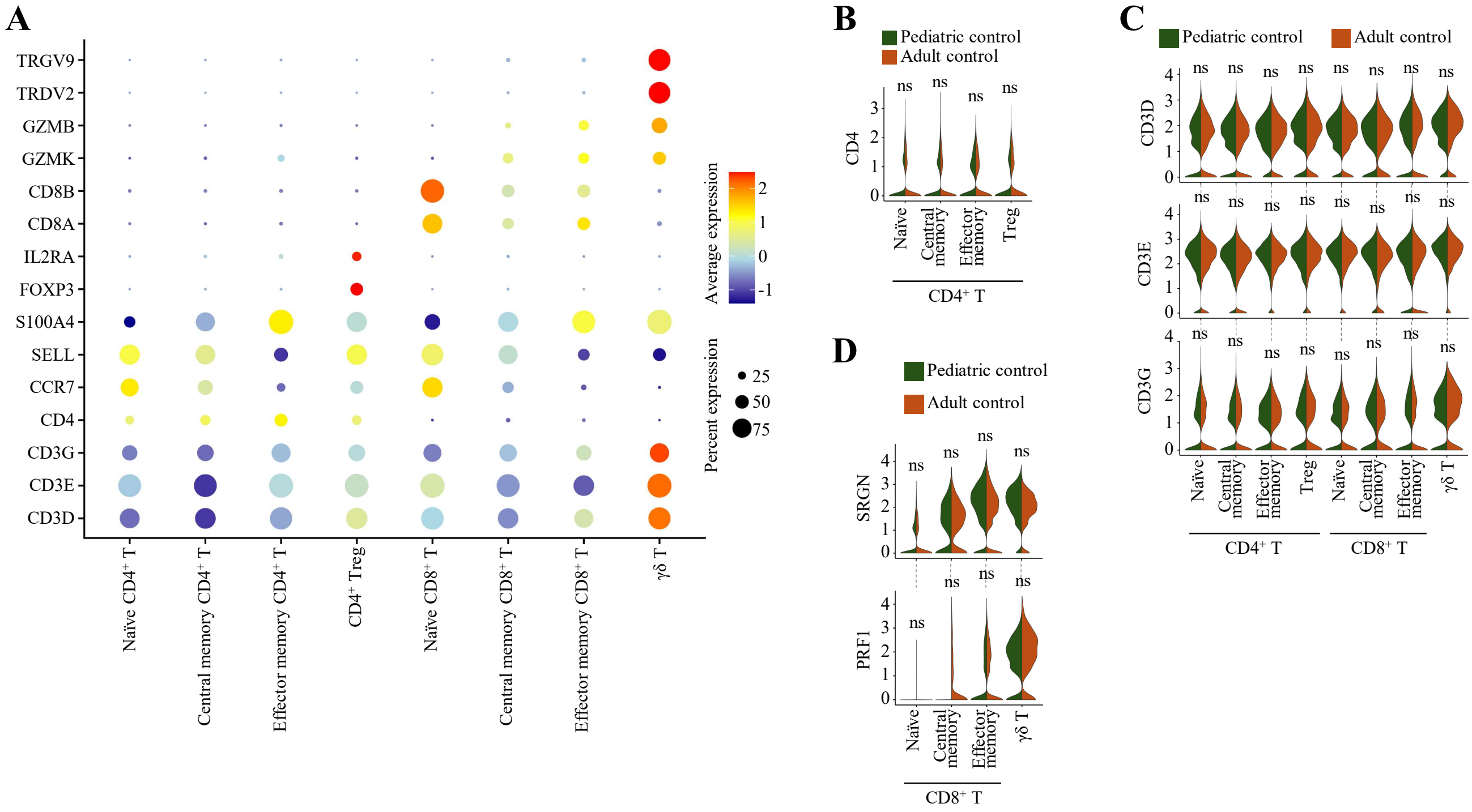

Figure S2. Validation of T cell subset classification and expression of receptor complex and cytotoxicity-related genes.

A. Dot plot showing the expression of canonical marker genes used to identify major T cell subsets, including CD3D, CD3E, CD3G, CD4, CCR7, SELL, S100A4, FOXP3, IL2RA, CD8A, CD8B, GZMK, GZMB, TRDV2, and TRGV9. Dot size represents the percentage of cells expressing each gene, and color indicates average expression level.

B. Violin plots showing expression of the T cell receptor complex gene CD4 in $CD4^+$ T cell subsets from pediatric and adult controls.

C. Violin plots showing expression of T cell receptor complex genes CD3D, CD3E, and CD3G across $CD4^+$, $CD8^+$, and γδ T cell subsets in pediatric and adult controls.

D. Violin plots showing expression of cytotoxicity-related genes SRGN and PRF1, which are associated with the granzyme-mediated programmed cell death pathway, in cytotoxic T cell subsets ($CD8^+$ T cells and γδ T cells) from pediatric and adult controls.

Gene expression differences in (B)–(D) were assessed using the Seurat FindMarkers() function with the Wilcoxon rank-sum test. *p* values were adjusted for multiple testing using the Benjamini–Hochberg method, and adjusted *p* values are reported.

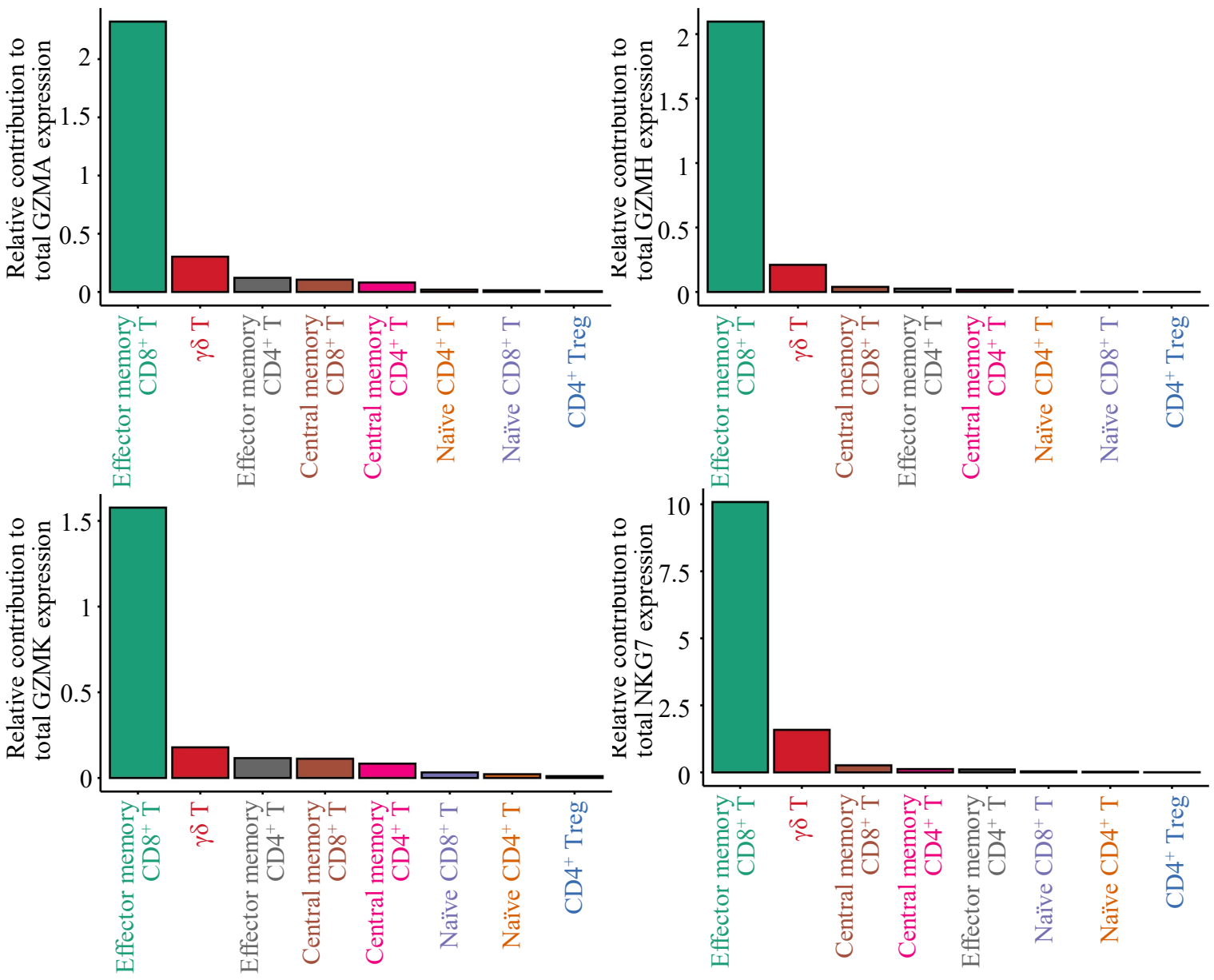

Figure S3. Relative contribution of T cell subsets to cytotoxic gene expression.

Bar plots showing the relative contribution of each T cell subset to total expression of *GZMA, GZMH, GZMK* and *NKG7*. Contribution was defined as the product of mean expression per cell within each subset and the proportional abundance of that subset among total T cells. 3

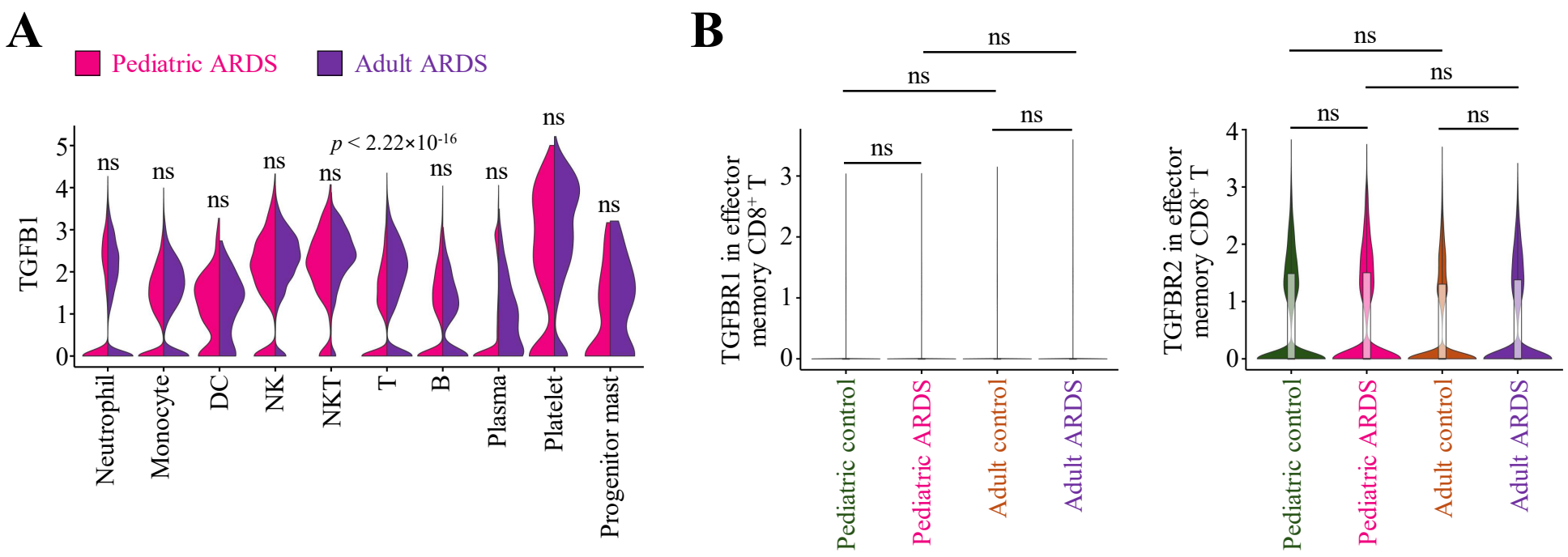

Figure S4. Single-cell TGFB1 and receptor expression across immune populations.

A. Violin plots showing single-cell TGFB1 expression across major immune cell populations (neutrophils, monocytes, dendritic cells, NK cells, NKT cells, T cells, B cells, plasma cells, platelets, and progenitor mast cells) comparing pediatric ARDS and adult ARDS. Each violin is split by group color.

B. Violin plots showing the expression of *TGFBR1*, and *TGFBR2* in $CD8^+$ effector memory T cells from pediatric controls, pediatric ARDS, adult controls, and adult ARDS.

Gene expression differences in (A) and (B) were assessed using the Seurat FindMarkers() function with the Wilcoxon rank-sum test. p values were adjusted for multiple testing using the Benjamini–Hochberg method, and adjusted p values are reported.

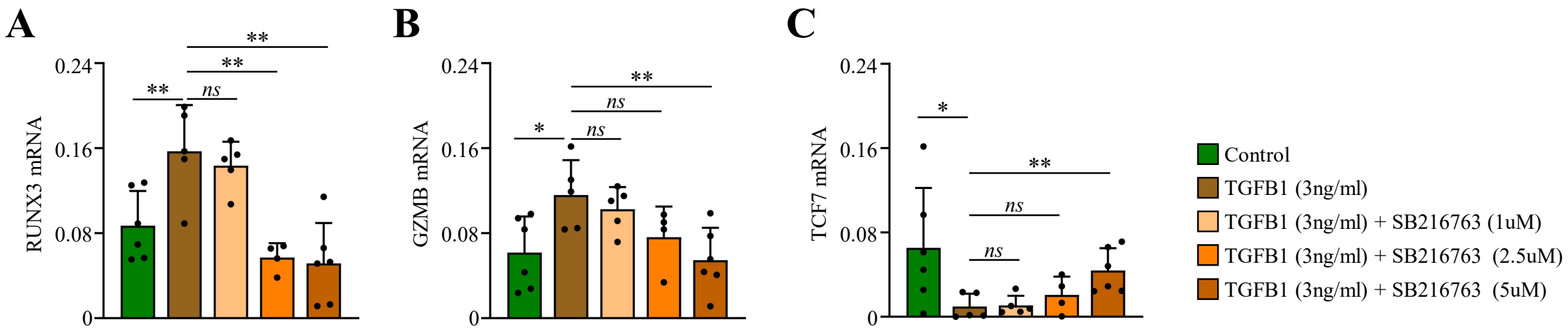

Figure S5. Dose-dependent effects of SB216763 on TGFB1-induced transcriptional changes in CD8$^+$ effector memory T cells

A–C. Sorted CD8$^+$ effector memory T cells were stimulated with TGFB1 (3 ng/mL) and simultaneously treated with increasing concentrations of SB216763 (1, 2.5, and 5 μM). The expression levels of GZMB (A), RUNX3 (B), and TCF7 (C) were measured by qPCR. Data are presented as mean ± SD and compared using an unpaired one-tailed Student's *t* test. *$p < 0.05$, **$p < 0.01$.

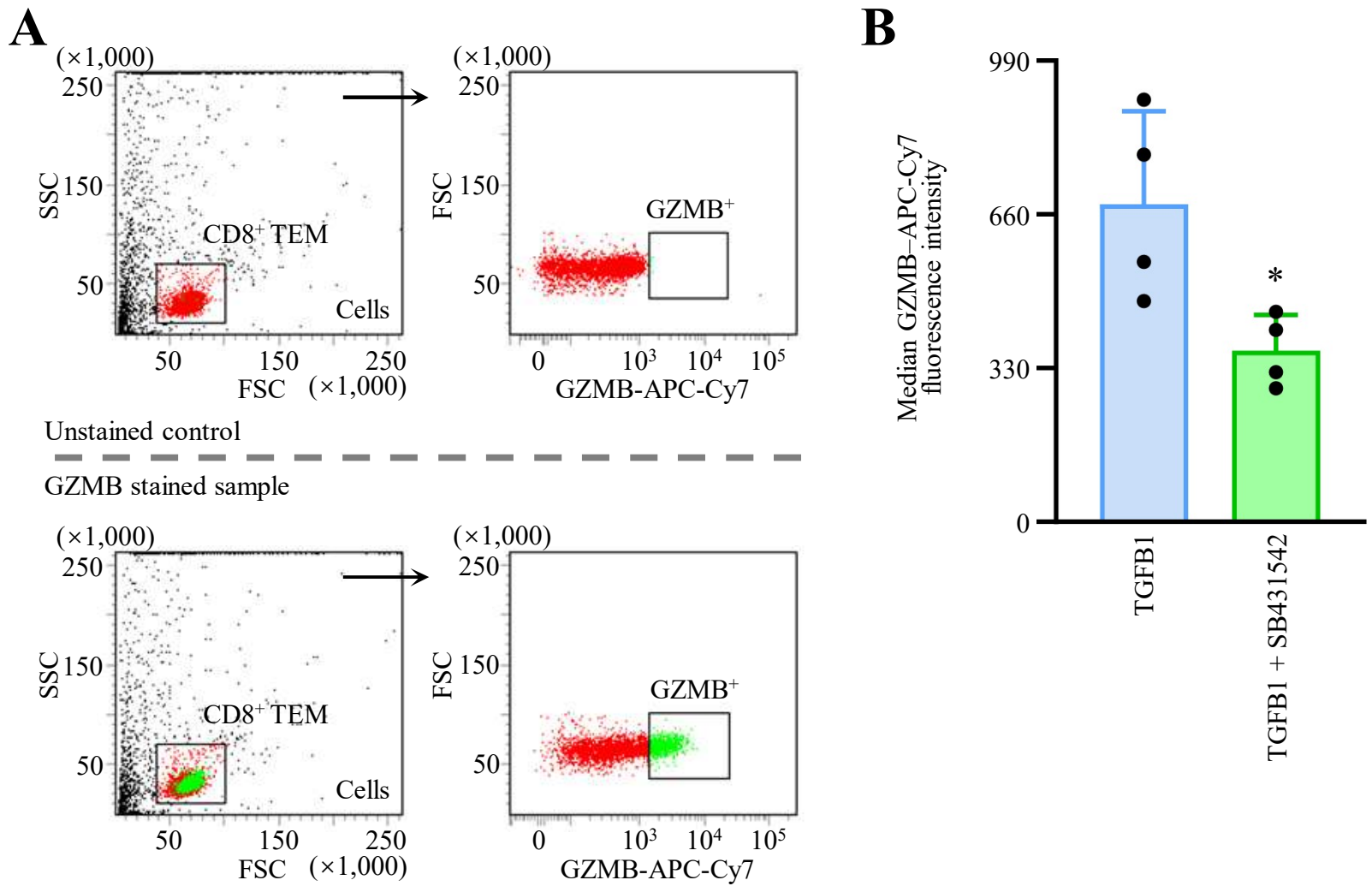

Figure S6. Flow cytometric analysis of GZMB expression in sorted CD8^{+} effector memory T cells.

A. Representative flow cytometry gating strategy for identification of GZMB-positive cells in sorted CD8^{+} effector memory T cells (TEM; CD3^{+}CD8^{+}CCR7^{-}CD45RA^{-}). Top panels show unstained controls used to determine background fluorescence. Bottom panels show GZMB-stained samples, with GZMB^{+} cells identified based on APC-Cy7 fluorescence.

B. Quantification of GZMB expression following TGFB1 stimulation with or without SB431542 treatment. GZMB expression was measured by flow cytometry and presented as median GZMB-APC-Cy7 fluorescence intensity.

Data in (B) are presented as mean ± SD and compared using an unpaired one-tailed Student's t test. *$p < 0.05$.

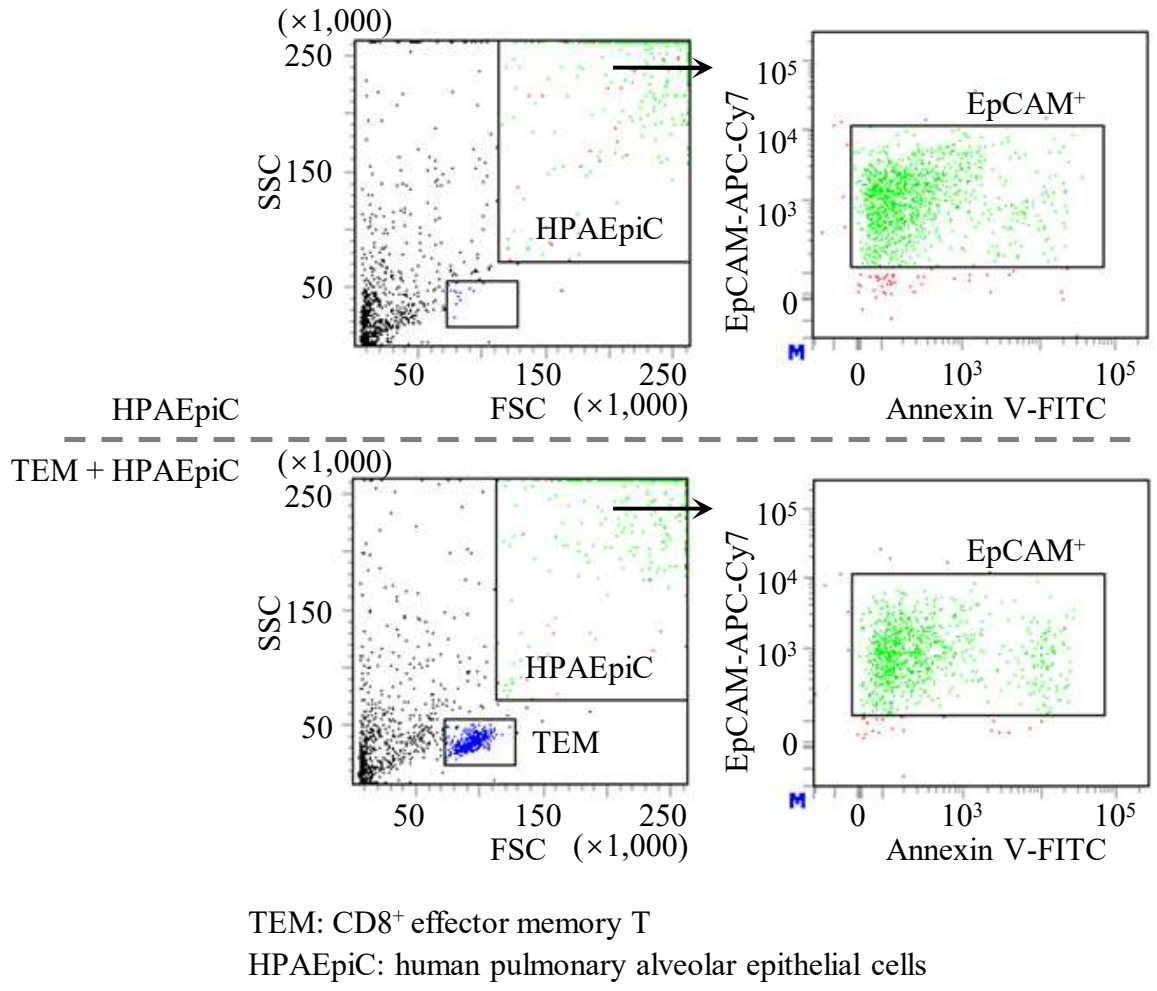

Figure S7. Flow cytometric gating strategy for identification of HPAEpiCs in CD8$^+$ effector memory T–HPAEpiC coculture assays.

Representative flow cytometry plots showing identification of human pulmonary alveolar epithelial cells (HPAEpiCs) in monoculture and after coculture with CD8$^+$ effector memory T cells. Based on FSC/SSC characteristics, epithelial cells were initially distinguished from other cell populations. EpCAM-APC/Cy7 staining was subsequently used to further confirm the HPAEpiC population in the coculture system.
